# Mycotoxins: A comprehensive review of its global trends in major cereals, advancements in chromatographic detections and future prospectives

**DOI:** 10.1016/j.fochx.2025.102350

**Published:** 2025-03-13

**Authors:** Waqas Niaz, Shahzad Z. Iqbal, Khurshid Ahmad, Abdul Majid, Waqas Haider, Xianguo Li

**Affiliations:** a Key Laboratory of Marine Chemstry Theory and Technology, Ministry of Education, Ocean University of China, Qingdao 266100, China; b Department of Applied Chemistry, Government College University, Faisalabad 38000, Pakistan; c State Key Laboratory of Marine Food Processing & Safety Control, College of Food Science and Engineering, Ocean University of China, No.1299, Sansha Road, Qingdao, Shandong Province, 266404, PR China; d College of Chemistry and Chemical Engineering, College of Life Sciences, College of Materials Science and Engineering, Institute of Biomedical Materials and Engineering, Qingdao University, Qingdao, Shandong Province 10 266404, PR China

**Keywords:** Mycotoxins, Occurrence, Chromatography, Cereals, Future prospectives, Food safety

## Abstract

Mycotoxins in commonly consumed cereals pose a significant global threat to human health and economic stability. This review examines the occurrence of key mycotoxins, specifically aflatoxins (AFTs), ochratoxin A (OTA), zearalenone (ZEN), deoxynivalenol (DON), and fumonisins (FUMs) in the most widely consumed cereals: corn, wheat, and rice. The results indicate substantial regional variations, with the highest contamination levels occurring in Africa (36.59 %), Asia (33.06 %), Europe (29.06 %), and the Americas (19.20 %). Among the mycotoxins, DON exhibited the highest average contamination rate across all regions, at 49.6 %. In Africa, AFTs (63.19 %) and fumonisins (60.17 %) were particularly prevalent. Significant contamination levels of DON (55.25 %) and OTA (47.24 %) were noted in Asia. Recent advancements in liquid chromatography-tandem mass spectrometry have enhanced the sensitivity and efficiency of detection methods. This review aligns with the overarching objective of safeguarding human health from the increasing risk of mycotoxins and ensuring a sustainable food and feed supply chain.

## Highlights

• Evaluation of most frequently found mycotoxins in cereals worldwide and human health impacts.
• African cereal contamination was 36.59 %, followed by Asia (33.06 %), Europe (29.06 %), America (19.46 %).
• Significant exploration of mycotoxin extraction, cleanup, and solvents used for their detection.
• Critical evaluation of chromatographic technique for mycotoxins analysis in cereals.

## Graphical abstract

**Figure:**
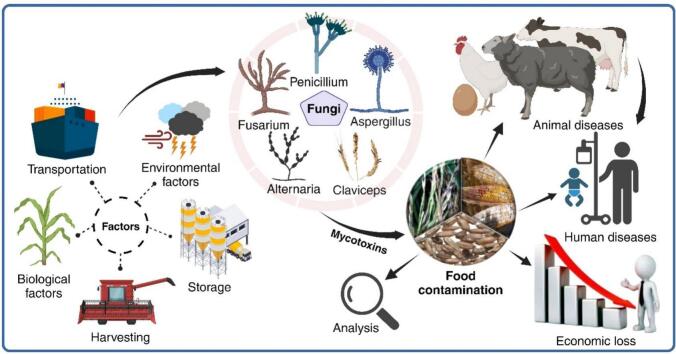

## List of abbreviation

**Table:** 

AFB_1_	Aflatoxins B_1_
AFB_2_	Aflatoxin B_2_
AFG_1_	Aflatoxin G_1_
AFG_2_	Aflatoxin G_2_
AFs	Aflatoxins
AFTs	Sum of aflatoxins B_1_, B_2_, B_3_, and B_4_
AhR	Aryl hydrogen receptor
AOAC	Association of Official Analytical Chemists
APCI	Atmospheric pressure chemical ionization
ASE	Accelerated solvent extraction
BEN	Balkan endemic nephropathy
DAD	Diode array detector
DES	Deep eutectic solvents
DON	Deoxynivalenol
DSPE	Dispersive solid phase extraction
EC	European commission
ECD	Electron Capture Detector
ELISA	Enzyme-linked immunosorbent assay
ESI	Electrospray ionization
EtOAc	Extraction solvents, such as a combination of ethyl acetate
EU	European Union
FA	Fumonisins A
FAO	Food and Agriculture Organization
FB_1_	Fumonisins B_1_
FB_1_B_2_	Fumonisins B_1_ & B_2_
FB_2_	Fumonisins B_2_
FB_3_	Fumonisins B_3_
FB_4_	Fumonisins B_4_
FBs	Fumonisins B
FCs	Fumonisins C
FDA	Food and Drug Administration
FHB	Fusarium head blight
FID	Flame Ionization Detector
FLD	Fluorescence
FPs	Fumonisins P
FUMs	Fumonisins
GC	Gas chromatography
HepG2	Human liver carcinoma
HCC	Hepatocellular carcinoma
HLB	Hydrophilic-lipophilic balance
HPLC	High-performance liquid chromatography
HPTLC	High-performance thin-layer chromatography
HQ	Hazard quotient
IAC	Immunoaffinity columns
IARC	International Agency for Research on Cancer
IMSPE	Immunomagnetic solid phase extraction
ISO	International Organization for Standardization
LC-MS	Liquid chromatography-tandem mass spectrometry
LLE	Liquid-liquid extraction
LOD	Limit of detection
LOQ	Limit of quantification
MAE	Microwave-assisted extraction
MLs	Maximum limits
mmt	Million metric tons
MRM	Multiple reaction monitoring
MNPs	Magnetic nanoparticles
MWCNTs	Multiwall carbon nanotubes
NRU	Neutral red uptake
OECD	Organization for Economic Co-operation and Development
OTA	Ochratoxin A
PDA	Photodiode array
PLE	Pressurized liquid extraction
PSQCA	Pakistan Standards and Quality Control Authority
QA	Quality assurance
QC	Quality control
QeOrbitrap	Quadrupole orbital ion trap
QLIT	Quadrupole linear ion trap
QqQ	Quadrupole
QuEChERS	Quick, easy, cheap, effective, rugged, and safe
PLE	Pressurized liquid extraction
SD	Standard deviations
ROS	Reactive oxygen species
SFE	Supercritical fluid extraction
SLE	Solid-liquid extraction
SPE	Solid phase extraction
TFU	Sum of fumonisins
TOF	Time-of-flight
UV	UV–visible
VADS-ME	Vortex-assisted liquid-liquid dispersive microextraction
VOS	Visualization of similarities
WHO	World Health Organization
ZEN	Zearalenone

## 1 Introduction

Mycotoxins are secondary fungal metabolites, a substantial worldwide concern with profound implications for human/animal health and economic stability (Azam et al., 2021; Kamle et al., 2022). So far, 400 types of mycotoxins have been recognized; nonetheless, aflatoxins (AFs), ochratoxin (OTA), zearalenone (ZEN), deoxynivalenol (DON), and fumonisins (FUMs) are most frequently found mycotoxins in cereals and cereal-based products (Abednatanzi et al., 2020; Palumbo et al., 2020). Mycotoxins fungi often infiltrate the crops and other food commodities during storage, transportation, and pre-and post-harvest conditions (Luo et al., 2018). Toxic fungi, including *Aspergillus*, *Penicillium*, *Fusarium*, and *Alternaria spp*, are the prominent contributors to mould production that cause crop contamination with mycotoxins (Majeed et al., 2018). For instance, field fungi, comprising *Fusarium* and *Alteraria spp*, cause the contamination of cereal grains under field conditions; similarly, when the grains are stored, they are invaded by *Penicillium* and *Aspergillus spp* (Mohapatra et al., 2017). Thus, the contamination of food or the food supply chain by various moulds is a potential issue.

**Fig. 1:**
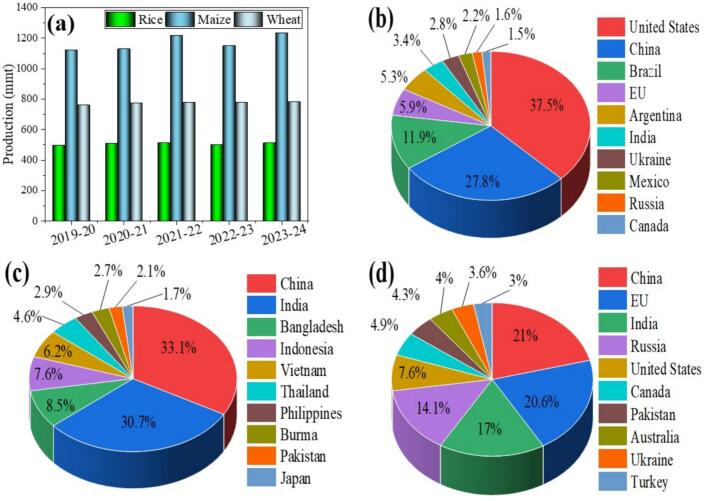
**a)** Global production of corn, rice, and wheat in last five years (million metric tons); **b**) Top 10 corn; **c)** rice; **d)** and wheat-producing countries as of 2024, adapted and redrawn from (https://www.statista.com and https://fas.usda.gov).

Maize (*Zea mays L.*), wheat (*Triticum aestivum*), and rice (*Oryza sativa*) are recognized as crucial food crops and serve as the main source of staple food in the world. Globally, in the past five years, the production of corn, rice, and wheat in a million metric tons (mmt) is shown in Fig. 1a. In addition, the top ten corn, rice, and wheat-producing countries are shown in Fig. 1b, Fig. 1c, and Fig. 1d respectively. Furthermore, the USA is the top corn-producing country, with China and Brazil ranking second and third, respectively (Fig. 1b). Moreover, China is the foremost rice-producing country, followed by India and Bangladesh, which are in second and third place, respectively. In addition, China is also first in wheat production, with the European Union (EU) and India following closely after (Fig. 1d). Worldwide, cereals occupy 700 million hectares of arable land, providing around 40 % of the energy and protein to the consumers (Dunwell, 2014). Mycotoxin contamination in cereals and food commodities is more prevalent in developing countries because of their weather, inadequate methods of cultivation, and improper management (Al-Jaal et al., 2019). Furthermore, cereals are the primary energy source for more than 60 % of the population in this developing country (Abate et al., 2022). According to the United Nations Food and Agriculture Organization (FAO), mycotoxins have been identified in over a quarter of cereal crops worldwide. This contamination causes a yearly loss of around one billion tons of food products/feed (Gab-Allah, Choi, & Kim, 2022; Lijalem et al., 2022; Romera et al., 2018). In contrast to the 25 % estimate given by the FAO, Eskola et al. (2020) reported that mycotoxins have been identified in around 60 % to 80 % of the world's grains. In the US, Ontario, and Canada, during 2018, approximately 63.5 mmt of grains were estimated to be contaminated with mycotoxins due to humidity and delayed harvesting factors. Furthermore, the anticipated average economic loss due to reduced yields caused by maize between 2016 and 2019 was calculated at US $138.13 per hectare (Mueller et al., 2020).

**Fig. 2:**
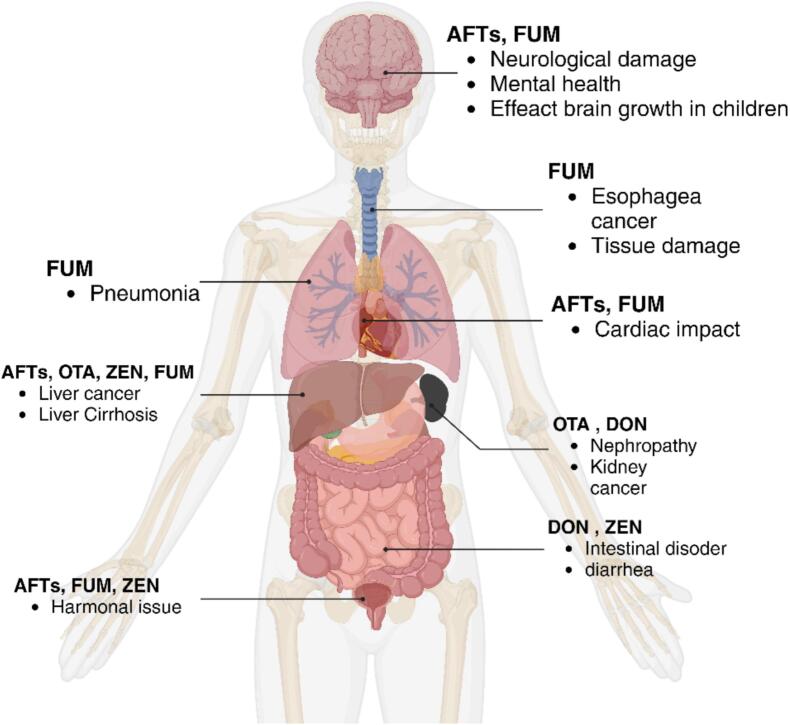
Effect of commonly found mycotoxins in cereals and impact on human health. **Note:** AFTs, Aflatoxins total; DON, Deoxynivalenol; ZEN, Zearalenone; FUM, Fumonisins; OTA, Ochratoxin A (image created with https://www.biorender.com).

Consumption of food contaminated (above the suggested level) with mycotoxins has posed a severe risk to human and animal health for decades (Gao et al., 2020). Mycotoxins can be ingested directly; however, that is not always the case; sometimes, they can be indirectly (by eating animals affected by mycotoxins). Mycotoxins can lead to a condition known as mycotoxicosis and have devastating effects on human and animal health, including haemorrhaging, nephrotoxicity, neurotoxicity, erogenicity, teratogenicity, immunosuppression, mutation, and carcinogenesis (Ben Taheur et al., 2019). Fig. 2 illustrates the influence of the most often encountered mycotoxins and their consequences on human health. Government agencies on both the national and international levels have worked together to address the issue of mycotoxin intoxication in food or the food supply chain. The World Health Organization (WHO), the European Union (EU), the Food and Agriculture Organization of the United Nations (FAO), and the US Food and Drug Administration (FDA) have all developed regulatory limits for the main classes of mycotoxins as well as for certain individual mycotoxins. According to the European Commission (European Commission, 2023), the recommended maximum limits (MLs) in cereals or cereals derived products are set at 2 μg/kg aflatoxins B_1_ (AFB_1_) and 4 μg/kg for the sum of aflatoxins (AFTs) B_1_, B_2_, B_3_, and B_4_ (AFTs) 4 μg/kg respectively. For OTA, MLs in unprocessed cereals or products derived from unprocessed cereal is set at (3–5 μg/kg). Similarly, for unprocessed cereal and maize grains, the MLs set for DON are 1250 μg/kg and 1750 μg/kg. In comparison, the allowable level of ZEN is 100 μg/kg (excluding unprocessed maize) for unprocessed cereals and 350 μg/kg for unprocessed maize grains. The MLs for the sum of fumonisins B_1_ & B_2_ (FB_1_B_2_) in unprocessed maize grains and maize placed to market for the end consumer are 4000 μg/kg and 1000 μg/kg, respectively. The International Agency for Research on Cancer (IARC) of the WHO has classified mycotoxins as carcinogenic for humans. The entire production cycle, from regulatory legislation to pre-harvest, harvest, and post-harvest management, must be synchronized to minimize fungal infections and degradation of mycotoxins. While several advanced economies have set maximum levels of mycotoxins allowed in food, other developing nations either do not have similar laws or have not implemented them well enough to prevent the exposure of cereal products to mycotoxins.

Although much focus has been given to detecting and analyzing mycotoxins in cereal products, several control measures and preventative methods have been used to reduce mycotoxin contamination during both pre-harvest and post-harvest stages. These techniques include implementing effective agricultural practices, improving storage conditions in order to inhibit fungal growth, and utilizing biological control technologies, all of which aid in decreasing mycotoxin levels in food sources. Nevertheless, food contamination is still a problem, underscoring the necessity of ongoing improvements in regulatory frameworks and detection technology to protect food safety. Thus, this review aims to update the systematic review of the global contamination level of mycotoxins in cereals (most widely consumed, i.e., corn, rice, and wheat) from 2019 to 2024 collected from various regions, including Asia, Africa, America, and Europe. Secondly, the most used chromatography methods for mycotoxin detection in cereal and their advantages and disadvantages were reviewed. Furthermore, there was extensive discussion of human health concerns and analytical technique performance processes, including the processes for cleanup and extraction. Pursuing these goals empowers scientists with an enhanced and cutting-edge framework and lays a solid groundwork for directing subsequent investigations.

## 2 Search methodology and scientometric analysis

**Fig. 3:**
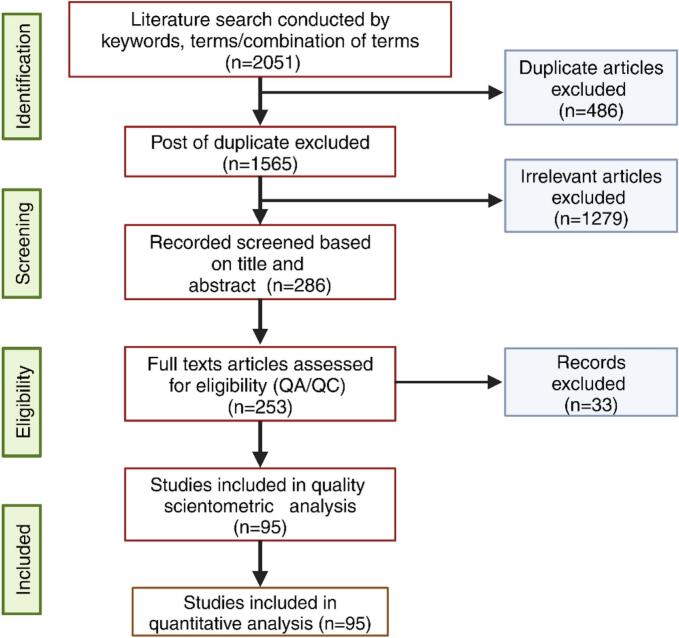
Flow sheet diagram of literature search and scientometric evaluation of chosen material.

All procedures and standards outlined by (Siddaway et al., 2019) and (Moher et al., 2010) have been followed in the present literature review, as depicted in Fig. 3. To examine the prevalence of significant mycotoxins in wheat, rice, and maize, as well as their associated health concerns and the role of chromatographic techniques in their detection, relevant peer-reviewed articles were selected using various search engines, including Google Scholar, Science Direct, Web of Science, and PubMed Scopus. This search was conducted based on the following combinations of terms “Mycotoxins Occurrence,” “Aflatoxins cereals mycotoxins,” “Ochratoxins cereals mycotoxins,” “Zearalenone cereals mycotoxins,” “Deoxynivalenol cereals mycotoxins,” “Nivalenol cereals mycotoxins,” Maize mycotoxins,” “Wheat mycotoxins,” “Rice mycotoxins,” “Occurrence mycotoxins cereals,“ and “Chromatography mycotoxins cereals.” The literature survey was limited to research published in English from 2019 to 2024. Using various search engines, 2051 results were obtained; 1665 papers met the criteria and were consequently selected for further analysis. Subsequently, we examined the abstracts and eliminated some that were irrelevant. Our final selection criteria were as follows: the study had to be original and cross-sectional, the entire manuscript had to be readily accessible, the mycotoxin prevalence or concentration in cereals had to be reported, and the analytical methods employed had to be defined. The worldwide occurrence or chromatographic detection techniques for wheat, rice, and maize mycotoxins were demonstrated using 95 references post quality assurance/quality control (QA/QC). Each reference created data tables and Figures, including mycotoxins type, first author name, sampling location, year of publication, number of total and positive samples, mycotoxins concentration ranges, sample source, solvent ratio matrix type (wheat, rice, maize), the limit of detection (LOD), the limit of quantification (LOQ), and chromatographic methods. Thus, our presentation contains detailed explanations, figures, and data tables, offering a comprehensive analysis of mycotoxin contamination in cereals, human health impact, and the most frequently used chromatographic technique.

**Fig. 4:**
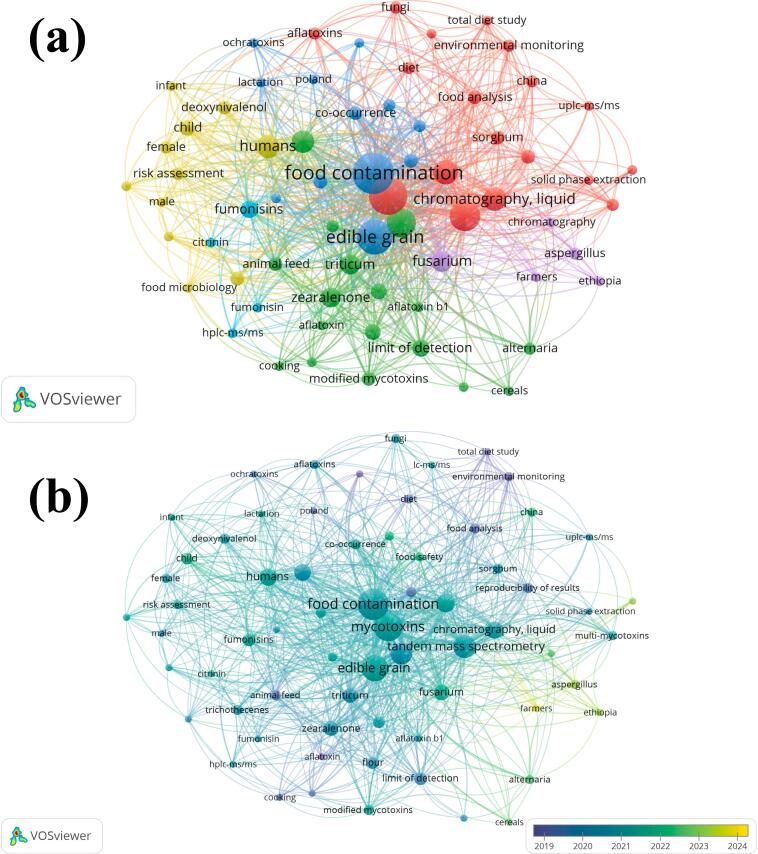
**a**) Network visualization of food mycotoxins studies based on text data retrieved from PubMed. Dots indicate a keyword component, with the size of the dot corresponding to the frequency of the word appearing in papers linked to mycotoxins published from 2019 to 2024. The linkage between dots signifies the level of correlation between two keywords: **b)** Overlay keywords visualization from the PubMed database pertaining to food mycotoxins from 2019 to 2024. This analysis sheds light on the prevailing themes, interconnections, and cutting-edge approaches in food mycotoxins by examining the literature on the occurrence of mycotoxins in cereals.

VOS (visualization of similarities) viewer efficiently revealed bibliometric networks to uncover the frequency and relationships between keywords and authors in academic literature. Advanced algorithms visually represent the current academic trends by visualizing complex relationships. An exhaustive inquiry was conducted utilizing the PubMed database with the keywords “Mycotoxins,” “Cereals,” “Occurrence,” and “Chromatography” from 2019 to 2024, resulting in the identification of over 46 research publications in this field. Using VOS viewer for a comprehensive examination of literature obtained from PubMed resulted in the creation of an intriguing thematic map that depicts the relationships among indexed terms. Visualizations like Fig. 4(a-b) illustrate 762 interrelated keywords associated with the emergence of these four keywords and main points throughout different periods. In the yearly publishing year chart, various colours visualize distinct year-wise data according to selected timeframes Fig. 4b. In density visualization, a deeper shade indicates a greater density. This clearly underscored the significance and emergence of mycotoxins in cereals, including different aspects such as food contamination, edible grain, co-occurrence, risk assessment, chromatography, effects on human health, food security, food analysis, and several others.

## 3 Common mycotoxins in cereals and health risks associated with mycotoxins

### 3.1 Mycotoxins in cereals

Multiple researchers have extensively examined and recorded the main classes of mycotoxins, including AFs, OTA, ZEN, DON, and FUMs in cereals and cereal-based products, which are discussed below.

#### 3.1.1 Aflatoxins

**Fig. 5:**
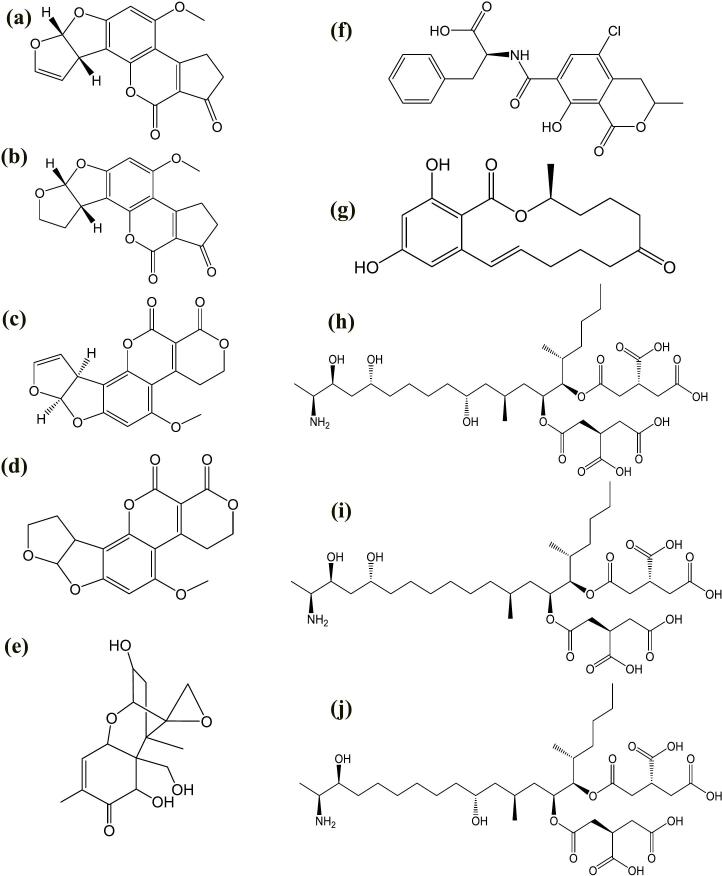
Chemical structure of most frequently found mycotoxins in cereals and cereals-based products **a)** Aflatoxin B_1_, **b)** Aflatoxin B_2_, **c)** Aflatoxin G_1_, **d)** Aflatoxin G_2_, **e)** Deoxynivalenol, **f)** Ochratoxin A, **g)** Zearalenone, **h)** Fumonisins B_1_, **i)** Fumonisins B_2_, **j)** Fumonisins B_3_.

*Aspergillus flavus* and *A. parasiticus* are considered to be the primary sources of AFs. *A. flavus* is the first discovered class known to be a significant source for producing B-type AFs, while *A. parasiticus* is responsible for producing G-type AFs. *A. ochraceous*, *A. pseudotamarii*, *A. parvisclerotigenus*, *A. bombycis,* and *E. mericella astellata* are all accountable for AFs production in food (Ahmad et al., 2014). Wheat, maize, rice, peanuts and barley are only a few kinds of cereals that host the AFs, including aflatoxin B_1_ (AFB_1_, C_17_H_12_O_6_), aflatoxin B_2_ (AFB_2_, C_17_H_14_O_6_), aflatoxin G_1_ (AFG_1_, C_17_H_12_O_7_), and aflatoxin G_2_ (AFG_2_, C_17_H_14_O_7_). So far, more than 20 types of AFs have been discovered (Kumar et al., 2017). The chemical structure of the most frequently found mycotoxins in cereals is depicted in Fig. 5. AFs can enter the body through the digestive system and damage the internal organs of humans/animals if they eat contaminated food or use items from infected animals. Prolonged and regular exposure to AFs has detrimental effects on the health of both humans and animals. The International Agency for Research on Cancer (IARC) classifies AFB_1_ as a group-I substance due to its toxicity, mutagenicity, immunotoxicity, teratogenicity, and carcinogenicity IARC (Marchese et al., 2018). Hepatocellular carcinoma (HCC), the most widespread liver cancer, causes mortalities of around 0.6 million people annually. AFB_1_ exposure is associated with 4.6 %–28.2 % of hepatocellular HCC cases (Kucukcakan & Hayrulai-Musliu, 2015). AFB_1_ is also a well-known carcinogen linked to animal and human kidney, lung, and colon cancers. Williams et al. (2004) reported that approximately 4.5 billion people in developing countries are widely affected by a single type of mycotoxins, namely AFs. A sum of aflatoxins (AFTs) in cereals is limited by the European Union (EU) to no more than 4 μg/Kg (EC 2023), significantly lower than the limits set by the Food and Drug Administration (FDA) of the United States (≤ 20 μg/Kg) (FDA 2020; Kaale et al., 2020).

#### 3.1.2 Ochratoxins A

In 1965, OTA, molecular formula (C_20_H_18_ClNO_6_), and molecular weight (403.813 g/mol) were first chemically studied after being isolated from cornmeal. OTA comprises a poisonous mycotoxin generated by the *Penicillium* and *Aspergillus* fungi (Bredenkamp et al., 1989). Most commonly, it is present in a wide variety of cereals and products made from cereals, including maize, wheat, barley, peanuts, rice, sorghum, and millet. Upon degradation, OTA produces several carcinogenic derivatives, including 14-(*R*)-ochratoxin A, ochratoxin- (OT), and ochratoxin amide (OT amide), all of which are milder toxins than parent OTA (Ryu et al., 2019). Frequent exposure to OTA in the human body causes various illnesses, including teratogenic and nephrotoxic effects. Cortical interstitial nephropathy is a kidney disease caused by OTA exposure that manifests; it also causes chronic interstitial nephritis (CIN), Balkan endemic nephropathy (BEN), and other similar diseases in humans. However, animal carcinogenicity studies have revealed a substance that may cause human cancer (Group 2B) (Echodu et al., 2019).

#### 3.1.3 Fumonisins

Gelderblom first identified FUMs from *F. moniliforme* in corn crops in South Africa (Gelderblom et al., 1988). Fungal pathogens of cereals, such as *Fusarium verticillioides* and *Fusarium proliferatum*, produce a class of secondary metabolites known as FUMs. Dozens of other FUMs have been discovered and named, but the most commonly found are Fumonisins A (FAs), Fumonisins B (FBs), Fumonisins C (FCs), and Fumonisins P (FPs). The three most common types of FBs are FB_1_, FB_2_, and FB_3_, with FB_1_ being the most hazardous and perhaps coexisting with the other two types. FBs cause the most common type of food contamination, and FB_1_ is the most common source of FBs; as much as 70 % of all FBs can be attributed to FB_1_ (Damiani et al., 2019). Two novel non-amino FUMs, Fumonisins Py and Fumonisins La, have been discovered mainly using the semi-target method. Compared to FBs, the novel Fumonisins Py and Fumonisins La are far less cytotoxic. This opens up new avenues of inquiry into the mechanisms by which FBs cause their harmful effects. Ingestion of FB_1_ effective foods has been related to several forms of cancer, including esophageal cancer. The IARC has classified FB_1_ as a Group 2B human carcinogen based on the available evidence. Embryos, digestive tracts, neurological systems, and the sex organs of men can all suffer damage from FB_1_ exposure (Liang et al., 2024).

#### 3.1.4 Deoxynivalenol

DON, also known as 12,13-epoxy-3,7,15-trihydroxy, trichothec-9-en-8-one, having a molecular formula of C_15_H_20_O_6_ and a molecular weight of 296.3 g/mol, is a class of secondary metabolites primarily created by *F. graminearum* and *F. culmorum* (Marin et al., 2013). Small grains grown in North America, Asia, and Europe commonly possess 16 phylogenetic species comprising the *F. sambucinum* and *F. incarnatum-equiseti* species complex. *F graminearum* is a pathogenic fungus originating from Fusarium head blight (FHB) in wheat and other grains (Yan et al., 2020). *F. culmorum* is commonly detected in grain roots, stem bases, and maize heads. The toxicological impact must be determined through extensive in vivo testing. The DON is sometimes also referred to as vomitoxin, as it causes nausea and vomiting in humans as well as animals. Humans who consume large amounts of DON may have gastroenteritis; likewise, in children, food contaminated with DON for an extended period may experience developmental delay and growth retardation (Luo et al., 2020).

#### 3.1.5 Zearalenone

ZEN, previously referred to as an F-2 toxin, is a resorcyclic acid lactone having a chemical formula of C_18_H_22_O_5_ and a molecular weight of 318.36 g/mol. Several species of *Fusarium* produce ZEN, an estrogenic mycotoxin, such as *F. graminearum*, *F. semitectum*, and *F. sporotrichioides* (Azam et al., 2021). When a consumer takes ZEN contaminated food, it can be detected in the blood, lungs, liver, bile, adipose, reproductive organs, breast milk, kidneys, and urine. In addition, recent research suggests that ZEN affects the liver of animals or humans. ZEN has been reported to have hepatotoxic, immunotoxin, carcinogenic, hormonal imbalances, and other harmful effects in animal tests (Rogowska et al., 2019). This type of mycotoxin also causes severe kidney toxicity by selecting contaminated food for a long duration (Ropejko & Twarużek, 2021).

### 3.2 Health risk assessment associated with mycotoxins in cereals

In Nepal, the AFs, OTA, DON, ZEN, and FUMs were the mycotoxins frequently found in maize samples. The highest contamination of maze samples with DON (100 %) and AFs (78 %) was reported. The predicted daily intake, margin of exposure, and liver cancer risk associated with maize eating were 30.46 ng/kg body weight (bw) day^−1^, 5.58, and 0.38 cancer cases per year per 100,000 individuals, respectively (Joshi et al., 2022). The occurrence of multiple mycotoxins (AFs, OTA, DON, ZEN, and FUMs) has also been reported in various cereals (wheat, rice, and corn flour) as well as in human urine in Brazil. The mean probable daily intake (PDI) values derived from occurrence data ranged from 0.007 to 0.013, 0.069 to 1.002, 0.119 to 0.321, and 0.013 to 0.156 μg/kg body weight (bw) day^−1^ for AFs, DON, FBs, and ZEN, respectively. The mean PDI values derived from urinary biomarkers were recorded as follows: 0.001, 84.914, 0.031, 0.377, and 0.002 μg/kg bw day^−1^ for AFB_1_, DON, OTA, FB_1_, and ZEN, respectively. The hazard quotient (HQ) using food data indicated a potential health concern regarding ZEN. HQ > 1, as indicated by urinary biomarkers, was also noted for DON. The HQ derived from urinary OTA levels exceeded 1. The margin of exposure values for AFs derived from food and urine data in the 1st SP were found to be below 10,000, suggesting potential health risks in the Brazilian population (Franco et al., 2019).

An investigation was conducted by Taghizadeh et al. (2020) to evaluate the risks for Iranians to assess the risks faced by Iranians from consuming contaminated rice containing various mycotoxins, including AFB_1_, OTA, and DON, in relation to both cancerous and non-cancerous outcomes. The estimated 95th and 50th centiles of Hazard Index (HI) in Iranians resulting from rice ingestion were found to be 2.5 and 0.5, respectively. The 95th and 50th centiles of individuals with positive surface antigens for hepatitis B (HBsAg^+^) risk characterization for AFB_1_ in Iranian rice consumers were 81 and 79.1, respectively. The 95th and 50th centiles for the risks associated with Iranians who are negative for the surface antigen of hepatitis B HBsAg (HBsAg^−^) were recorded at 4.4 and 2.6, respectively. The findings regarding cancer risk effects indicated that the margins of exposure (MOE) at the 95th and 50th centiles were 233 and 231, respectively.

A similar study was conducted by Adetunji et al. (2017) in different zones of Nigeria to assess the health risks in infants and young children (IYC) associated with contaminated maize grains. The PDI method was utilized to assess exposure to different mycotoxins (AFs, OTA, FUMs, ZEN and DON) while the MOE and the population at risk of primary hepatocellular carcinoma were employed to evaluate the risk associated with the consumption of AFs contaminated maize. IYC in the Derived Savannah zone faces higher exposure to AFs, OTA, and ZEN, whereas those in the Northern Guinea Savanna zone primarily encounter DON and FUMs. The average national MOE for IYC was recorded at 0.12 and 0.3, respectively. At the same time, the estimated risk of developing primary liver cancer stood at 152.7 and 61.1 cases per year per 100,000 population of IYC, respectively. A similar investigation was conducted by (Memar et al. (2024) in Iran to examine the occurrence of AFTs, AFB_1_, and OTA in imported rice samples and the associated health risks due to the consumption of rice contaminated with mycotoxins. The highest contamination rate of AFTs (24.18 %) in was reported in rice samples, followed by AFB1 (17.52 %) and OTA (1.07 %). The carcinogenic risk in Iran is reported to be higher, with projected risk percentages for AFB_1_ being 22.20 for adults and 43.50 for children. All these findings indicate that the consuming maize, wheat and rice contaminated with mycotoxins is at risk of experiencing adverse health effects.

## 4 Occurrence of mycotoxins in cereals

Collecting region-wise data (Asia, Europe, America, and Africa) has been a complex task, with each approach offering unique levels of sensitivity and accuracy. This diversity in data collection methods can pose a challenge when attempting to make quantitative comparisons.

### 4.1 Occurrence of mycotoxins in cereals (corn, rice, and wheat) in Asia

The Asian continent spans a vast extent of 44 million square kilometres. It has a unique continental monsoon climate and complicated geographical characteristics, resulting in favourable conditions for mould proliferation and metabolic activity that produce toxins (Fei et al., 2020; C. Liu & Van der Fels-Klerx, 2021). In line with FAO statistics from 2010, Asian cereal production comprises approximately 50 % of world cereal production, establishing it as one of the main regions that provide cereal food and feed worldwide (OECD/FAO, 2010).

China, located in East Asia, significantly contributes to the worldwide production of corn, rice, and wheat (X. Zhang et al., 2014). The concentrations of mycotoxins in cereals have been the subject of many investigations in China. For instance, Yang et al., (2019)investigated the prevalence of the most frequently found mycotoxins in maize and maize flour, such as AFB_1_, AFB_2_, AFG_1_, AFG_2_, FB_1_, FB_2_, FB_3_, DON, and ZEN. FB_1_ (48 %) was the most widespread mycotoxin, followed by DON (35 %) and ZEN (30 %). In Pakistan, FB_1_ was prevalent, occurring in 86.11 % wheat and 90.90 % wheat flour samples. The contamination levels ranged from 4 to 1560 μg/kg in wheat and 4–1390 μg/kg in wheat flour (Iqbal, Usman, et al., 2020). In another study of maize conducted by Gillani Ul et al. (2022), AFTs and OTA were reported with a positive rate of 69 % (14.50–92.36 μg/kg) and 61 % (0.1–56 μg/kg), respectively. In maize, AFTs and OTA surpass 54 % and 22 % limits set (20 μg/kg) by the Pakistan Standards and Quality Control Authority (PSQCA). The AFB_1_ contaminated level in rice samples was exceeded by 3 % of the sample limit set by the EU. The maximum concentration of AFB_1_ was analyzed in wheat flour at 3.1 μg/kg (Zahra et al., 2019). Similarly, another investigation conducted by (Iqbal, Usman, et al., 2020) showed that the DON level exceeded the limit set by the EU 2006 (2000 μg/kg) in 16.47 % of samples of wheat and corn. In another South Asian country (India), rice samples sourced from local shops reported a positive rate of AFB_1_ (54 %), AFB_2_ (34 %), and AFTs (54 %). The contamination level of these mycotoxins was within the range of (0–20.34 μg/kg), (0–1.54 μg/kg), and (0–21.89 μg/kg), respectively. In only three samples, the level of AFTs surpassed the threshold of 15 μg/kg set by the Indian government (Validandi et al., 2024).

During 2019–2024 (Table 1 and **Table S1)**, maize, rice, and wheat were more contaminated in the Asian region with AFTs types AFB_1_ (35.08 %), followed by AFB_2_ (17.09 %), AFG_1_ (6.48 %) and AFG_2_ (5.86 %). The highest contamination percentage of AFB_1_ was observed in maize, rice, and wheat in the following order: Maize (53 %) > Rice (31.36 %) > Wheat (20.66 %). Similarly, AFB_2_ was most frequently found mycotoxins in rice samples (34 %). In Asia, TFU and DON were the most commonly found mycotoxins in targeted cereals, with a contamination rate of 57.17 % and 55.24 %, respectively. The highest contamination of DON was observed in the wheat (68.32 %) and rice (61.62) samples. Among FUMs, FB_1_ was the most commonly found mycotoxin in maize samples, with a contamination rate of 66.90 %. The results underscore the immediate need for improved food safety protocols, comprehensive education for farmers, and strengthened regulatory systems to mitigate the health hazards linked to mycotoxin contamination in cereals across Asia.

**Table 1:** Occurrences of mycotoxins in Asia (cereals) over the past five years (2019-2024).

Mycotoxins	Matrix	Samples/Positive	Incidence (%)	Concentration Range (μg/kg)	Sample source	Country	Year	References
AFTs	Rice	27/50	54	n.d-21.89	Local shops	India	2024	(Validandi et al., 2024)
	Maize	107	50.5	3-1663	Municipalities	Philippines	2023	(Romero & Cumagun, 2023)
	Wheat flour	43/108	40	0.34–7.61	Flour factories	Iran		(Heshmati et al. 2023)
	Maize	35/45	78	1.52-91.24	Local market/shops, farmer	Nepal	2022	(Joshi et al., 2022)
	Maize	93/135	69	14.50-92.36	Fields & research stations	Pakistan		(Gillani et al., 2022)
	Rice	9/18	50	2.0-.8.1	Supermarkets	Palestine		(Salman & Mudalal, 2022)
	Wheat flour	7/17	41	2.0.8.1				
	Corn	86/665	13	n.d-482	Different sources	China	2020	(Ren, Qin and Guan, 2020)
AFB_1_	Rice	27/50	54	n.d-20.34	Local shops	India	2024	(Validandi et al., 2024)
	Wheat flour	65/108	60	0.14-7.34	Flour factories	Iran	2023	(Heshmati et al., 2023)
	Rice	19/192	9.8	0.2-3.3	Farms and Market	China	2022	(Hu et al., 2022)
		48/128	38	-	Retail markets	UAE		(Alwan et al., 2022)
	Wheat bran	54/60	90	0.06-0.99	Manufacturer	Iran	2021	(Jahanbakhsh et al., 2021)
	Wheat Flour	144/180	80	0.01-0.05				
	Wheat	166/300	55	1.05-7.36	Warehouse	Lebanon	2020	
	Rice	13/144	9	n.d-93	Retail market, Households	Viet Nam		(Do et al., 2020)
	Maize	57/189	30	n.d-1572				
	Wheat flour	10/30	33	n.d-3.01	Shops, domestic	Pakistan	2019	(Zahra et al., 2019)
AFB_2_	Rice	17/50	34	n.d-1.54	Local shops	India	2024	(Validandi et al., 2024)
DON	Maize & maize flour	3/15	20	n.d-11.83	Retail stores & supermarkets	Indonesia	2024	(Shantika et al., 2024)
	Wheat	81/103	78.6	25.1-7450	Fields	China	2023	(Dong et al., 2023)
	Maize	38/86	44.2	11.7-154				
	Wheat grain	307/321	95.6	n.d-8116				(Ji et al., 2023)
	Rice	12/12	100	0.08-0.34	Supermarkets			(Tan et al., 2023)
	Wheat	12/12	100	n.d-6.96				
	Rice	3/122	2.5	10.8-35.2	Fields			(Dong et al., 2023)
	Wheat flour	-	75	0.8-371.4	Flours markets		2022	(Zhou et al., 2022)
	Maize silage	199/200	100	n.d-3587	Dairy farms			(Zhang et al., 2022)
	Maize	45/45	100	110-520	Local markets, shops, farmers	Nepal		(Joshi et al., 2022)
	Wheat	910/1010	90.11	< 4 – 3070	Farmers	China	2022	(Li et al., 2022)
	Corn	539/1220	44.2	25–6660.5	Market, Farmers	Pakistan	2021	(Iqbal et al., 2021)
	Rice	-/2170	26.3	-	Fields	China	2020	(X. Wang et al., 2020)
	Wheat flour	-/10192	77.5	-	Fields			
	Wheat	13/50	26	58–1092	Factories and market	Turkey		(Golge & Kabak, 2020)
	Maize	63/100	63	-	Farmers and Local shops	Pakistan		(Raza, Asi and Maqbool, 2020)
	Wheat	579/579	100	12.16-6436.11	Farmers	China		(Yan et al., 2020)
	Maize	605/606	99.83	n.d-4300.7	Farmers	China		(Yan et al., 2020)
	Maize meal	-/1750	80.9	-	Fields	China		(Wang et al., 2020)
	Wheat	195/449	43.42	50-2145	Shops, Markets	Pakistan		(Iqbal, Usman, et al., 2020)
	Corn	144/270	53.33	50-2967	Shops, Markets			
	Maize	2/15	13	313–331	Factories and market	Turkey		(Golge & Kabak, 2020)
	Wheat	370/370	100	109.6–86255.1	Farmers	China	2019	(Xu et al., 2019)
	Maize & Maize flour	99/283	35	n.d-1570	Local market			(X. Yang et al., 2019)
FB_1_	Maize	30/20	67	1.89-3.86	Market	China	2022	(Wang et al., 2022)
	Wheat	30/20	67	1.81-3.42				
		41/1010	4.05	-	Farmers			(Li et al., 2022)
	Raw maize	47/58	81	n.d-24900	Farms and Markets			(Hu et al., 2022)
	Maize silage	144/200	72	n.d-558	Dairy farms			(Zhang et al., 2022)
	Wheat grain	31/36	86.11	4-1560	Supermarkets, stores, and vendors	Pakistan	2020	(Iqbal, Rehman, et al., 2020)
	Wheat flour	20/22	90.90	4-1390				
	Maize	63/189	33.33	n.d-1662	Retail markets, Households	Viet Nam		(Do et al., 2020)
	Maize & Maize flour	135/283	48	n.d-9845	Local markets	China	2019	(X. Yang et al., 2019)
	Maize/ maize-based products	47/58	81	n.d-24890	Farms and local markets			(Hu et al., 2019)
FB_2_	Maize silage	120/200	60	n.d-198	Dairy farms	China	2022	(Zhang et al., 2022)
	Maize & Maize flour	66/283	23	n.d-2307	Local markets		2019	(X. Yang et al., 2019)
	Maize/ maize-based products	36/58	62	n.d-5503	Farms and local markets			(Hu et al., 2019)
FB_3_	Maize silage	65/200	32	n.d-79.5	Dairy farms	China	2022	(Zhang et al., 2022)
	Maize & Maize flour	60/283	21	n.d-1090	Local markets		2019	(Yang, Gao, Liu and Yang, 2019)
TFU	Maize silage	755/910	83	n.d-38563	Dairy farms	China	2022	(Zhang et al., 2022)
	Maize	34/45	76	200-4180	Local market/shops, farmer	Nepal	2022	(Joshi et al., 2022)
	Corn	412/665	62	n.d-23480	Different sources	China	2020	(Ren et al., 2020)
	Wheat	21/72	29	n.d-710	Different sources			
	Corn flour	44/44	100	1000	Market	Japan		(Yoshinari et al., 2020)
	Corn snacks	66/66	100	45				
OTA	Maize	82/135	61	0.1-56.	Fields & research stations	Pakistan	2022	(Gillani et al., 2022)
	Maize	28/45	62	1.3.22	Local markets, shops and farmers	Nepal		(Joshi et al., 2022)
	Rice	2/10	20	0.3-5.6	Supermarkets	Palestine		(Salman & Mudalal, 2022)
	Maize	26/189	13.75	n.d-126	Retail market, Household	Viet Nam	2020	(Do et al., 2020)
	Wheat	145/300	48	0.51-9.71	Warehouses	Lebanon		(Joshi et al., 2022)
ZEN	Rice flour	2/10	20	0.8-2.8	Supermarkets and retail stores	Korea	2023	(Lijalem et al., 2023)
	Corn flour	21/24	88	0.5-536				
	Wheat flour	8/24	33	0.6-2.3				
	Wheat	111/26	49	n.d-619	Supermarkets	China		(Tan et al., 2023)
	Wheat grain	172/321	53.6	n.d-220	Fields			(Ji et al., 2023)
	Maize silage	719/910	79	n.d-10467	Dairy farms		2022	(Zhang et al., 2022)
	Wheat flour	-	40	0.2-5.7	Flours markets			(Zhou et al., 2022)
	Maize silage	159/200	69	n.d-832	Dairy farms			(Zhang et al., 2022)
	Maize	34/45	76	11.12-69.52	Local market/shops, farmer	Nepal		(Joshi et., 2022)
	Wheat flour	17/39	44	6.8-21.3	-	China	2020	(Hong et al., 2020)
	Maize	3/15	20	18–337	Factories and markets	Turkey		(Golge & Kabak, 2020)
	Corn	279/665	42	n.d-1307	Different sources	China		(Ren, Qin and Guan, 2020)
	Maize	36/189	19.04	n.d-212	Retail markets, Households	Viet Nam		(Do et al., 2020)
	Corn	28/40	70	3.2-743.2	-	China		(Hong et al., 2020)
	Wheat	30/72	42	n.d-116	Different sources			(Ren, Qin and Guan, 2020)
	Wheat	08/19	37	3.0-117.5	-			(Hong et al., 2020)
	Maize & Maize flour	84/283	30	n.d-721	Local markets		2019	(X. Yang et al., 2019)
	Wheat	254/370	69	0.3–1091.4	Farmers			(Xu et al., 2019)

**Note:** AFTs, Aflatoxins total; AFB_1_, Aflatoxin B_1_; AFB_2_, Aflatoxin B_2_; DON, Deoxynivalenol; ZEN, Zearalenone; FB_1_, Fumonisins B_1_; FB_2_, Fumonisins B_2_; OTA, Ochratoxin A; FB_1_B_2_, Sum of fumonisins B_1_ & B_2_; TFU, Total fumonisins.

### 4.2 Occurrence of mycotoxins in cereals (corn, rice, and wheat) in Europe

In Belgium, located in Western Europe, the prevalence rate of DON and ZEN in maize samples was reported to be 85.6 % and 49.8 %. Highest contamination levels reached 5322.4 μg/kg for DON and 2791.6 μg/kg for ZEN. In addition, only the ZEN level in 2.3 % of samples and 7.8 % of DON exceeded the EC allowable level (Vandicke et al., 2019). All samples of forage maize in Germany were found to be contaminated with DON, with a maximum value of 10,972 μg/kg. ZEN contamination was found in 96 % of samples with the highest 3910 μg/kg level. Only 9 % of samples had DON levels above the EC 2006 recommended limit (5000 μg/kg), while 46 % exceeded the ZEN recommended limit of 500 μg/kg in feedstuff (Birr et al., 2021). In Austria, a survey of silage maize from various dairy farms found DON to be the most prevalent mycotoxin (79 %), followed by FB_1_ (75 %), ZEN (61 %), and FB_2_ (50 %). DON contamination levels ranged from 30 to 1220 μg/kg, indicating a higher prevalence in maize silage (Penagos-Tabares et al., 2022). In Spain, maize samples were studied for different mycotoxins, including FB_1_, FB_2_, DON, ZEN, AFB_1_, AFB_2_, AFG_1_, AFG_2_, AFTs, and OTA. FB_1_ was the most frequently detected mycotoxin, found in 71.1 % of the samples, followed by FB_2_. AFB_1_ and AFB_2_ were present in 9.2 % of the samples, with 3 % exceeding the EU established limits for AFTs. Notably, no sample was reported to be contaminated with OTA (Tarazona et al., 2020).

AFB_2_ is significantly prevalent in maize samples from Europe, with some samples exhibiting an incidence rate of 100 %, the overall average incidence is 30.05 % (Table 2 and **Table S2**). AFB_2_ also shows significant contamination in maize, with an average of around 54.56 %. In contrast, rice samples display lower average contamination at 12.46 % compared to maize, suggesting that maize is more vulnerable to these mycotoxins. DON exhibits a significant presence in maize and rice, with some wheat samples also reporting a 100 % occurrence in Greece. This highlights the significant risk posed by DON in both maize and rice. The occurrence of OTA is relatively low across all cereal types, averaging 5.83 %. This suggests that while OTA remains a concern, its prevalence is notably less than that of other mycotoxins in the analyzed sample. ZEN exhibited considerable variability, with a relatively high average incidence of 31.79 % in maize. Conversely, its presence in wheat is minimal, indicating the differing levels of susceptibility among these crops.

**Table 2:** Occurrences of mycotoxins in Europe (cereals) over the past five years (2019-2024).

**Mycotoxins**	**Matrix**	**Samples/Positive**	**Incidence** **(%)**	**Concentration Range (μg/kg)**	**Sample source**	**Country**	**Year**	**References**
AFB_1_	Rice	2/9	22	n.d-1.62	Supermarkets	Spain	2022	(Romero-Sánchez, et al., 2022)
		41/9	22	0.17-1.60			2021	(Udovicki et al., 2021)
	Maize	32/15	47	0.28-28.15				
DON	Maize	3/15	20	n.d-1.79	Fields	Croatia	2024	(Zadravec et al., 2024)
	Maize	205/268	74	22-9923	Fields	Croatia	2023	(Janić Hajnal et al., 2023)
		38/400	10	50-752	Fields	Serbia		
	Wheat flour	9/75	12	110.6-607	Retail markets	Poland	2022	(Pokrzywa & Surma, 2022)
	Maize flour	8/24	33.3	129-1511				
		2/20	10	84.4-212				
	Maize silage	22/28	79	30-1220	Dairy farms	Austria		(Penagos-Tabares et al., 2022)
	Wheat	16/71	23	112-1916	Warehouses	Albania	2021	(Topi et al., 2021)
	Maize	120/120	100	n.d-10972	Farmers	Germany		(Birr et al., 2021)
		11/45	24	110-799	Warehouses	Albania		(Topi et al., 2021)
		31/98	31.6	n.d-1737	Grain stores	Spain	2020	(Tarazona et al., 2020)
		220/257	85.6	n-d-5322.4	Dairy farms	Belgium	2019	(Vandicke et al., 2019)
FB_1_	Maize	389/400	97	9-21239	Fields	Serbia	2023	(Janić Hajnal et al., 2023)
	Maize silage	21/28	75	14-356	Dairy farms	Austria	2022	(Penagos-Tabares et al., 2022)
	Maize	70/98	71.1	n.d-50.9	Grain store	Spain	2020	(Tarazona et al., 2020)
		74/257	28.6	n.d-4414.9	Dairy farms	Belgium	2019	(Vandicke et al., 2019)
	Maize flour	33/64	51.6	n.d-1.46	Supermarkets & retail shops	Hungry	2019	(Zentai et al., 2019)
FB_2_	Maize	388/400	97	5-5825	Fields	Serbia	2023	(Janić Hajnal et al., 2023)
	Maize silage	14/28	50	10.1-97.8	Dairy farms	Austria	2022	(Penagos-Tabares et al., 2022)
	Maize	55/98	56.1	n.d-12.1	Grain stores	Spain	2020	(Tarazona et al., 2020)
		26/257	10.2	n.d-1427.4	Dairy farms	Belgium	2019	(Vandicke et al., 2019)
FB_1_B_2_	Maize flour	3/12	25	160.8-216.4	Retail markets	Poland	2022	(Pokrzywa & Surma, 2022)
	Maize	34/45	76	59.9-16970	Warehouses	Albania	2021	(Topi et al., 2021)
TFU	Maize	170/191	89	24-13800	Fields	Croatia	2023	(Janić Hajnal et al., 2023)
		7/10	70	n.d-1009.36	Markets	Romania	2022	(Mihalcea & Amariei, 2022)
		73/257	28.6	n.d-6253.5	Dairy farms	Belgium	2019	(Vandicke et al., 2019)
OTA	Wheat grain	2/11	18	0.9-2.9	Markets	Poland	2019	(Hajok et al., 2019)
	Corn flour	3/45	7	0.7-1.6				
	Wheat flour	13/113	12	0.7-5.8				
ZEN	Maize	168/382	44	3.1-1241	Fields	Croatia	2023	(Janić Hajnal et al., 2023)
		128/257	49.8	n.d-2791.6	Dairy farms	Belgium	2019	(Vandicke et al., 2019)
	Maize silage	17/28	61	2.08-53.9		Austria	2022	(Penagos-Tabares et al., 2022)
	Maize	115/120	96	n.d-3910	Farmers	Germany	2021	(Birr et al., 2021)
		2/45	4.4	218-263	Warehouse	Albania	2021	(Topi et al., 2021)
		24/98	24.5	n.d-11.3	Grain store	Spain	2020	(Tarazona et al., 2020)

**Note:** AFB_1_, Aflatoxin B_1_; AFB_2_, Aflatoxin B_2_; AFTs, Aflatoxins total (AFB_1_, AFB_2_, AFG_1_, AFG_2_); DON, Deoxynivalenol; ZEN, Zearalenone; FB_1_, Fumonisins B_1_; FB_2_, Fumonisins B_2_; OTA, Ochratoxin A; FB_1_B_2_, Sum of fumonisins B_1_, & B_2_; TFU; Total fumonisins.

### 4.3 Occurrence of mycotoxins in cereals (corn, rice, and wheat) in America

The United States is a leading corn producer worldwide, vulnerable to mycotoxin contamination due to its distinctive features. In the USA, an assessment of corn grain and corn silage, mycotoxins AFB_1_, AFTs, OTA, TFU, DON, and ZEN found positive 5.47 %, 15.91 %, 2.73 %, 68 %, 83.31, and 19 %, respectively. TFU was the most prevalent mycotoxin, with the highest level of 59,117 μg/kg, and in 17.72 % of samples, detected levels were higher than 5000 μg/kg (Weaver et al., 2021). Another study in the USA indicates that DON, FB_1_, FB_2_, FB_3_, and ZEN were detected in 96.66 %, 88.4 %, 84 %, 80 %, and 84 % of maize grains, respectively (Fusilier et al., 2022). Mycotoxin contamination impacts a broad array of cereal grains in Brazil. The incidence of FB_1_ and FB_2_ in maize flour samples collected from retail stores was the same at 94 %. The concentration level of FB_1_ (21.1–2582 μg/kg) was higher as compared to FB_2_ (8–1148 μg/kg) and FB_3_ (7.5–757.6 μg/kg). After FUMs, DON was the most commonly found mycotoxin at 46 % in maize flour samples ranging from 3 to 595 μg/kg (Andrade et al., 2020). In another study in Brazil, ZEN was highly contaminated in 92.1 % of wild rice samples, and the concentration ranged from (7.5–757.6 μg/kg). Remarkably, 85 % of the samples exhibited ZEN levels surpassing 100 μg/kg (Tralamazza et al., 2021).

In Colombia, the AFs level was measurable in 75.6 % of rice samples collected from local stores and markets. AFB_1_ (71 %) was the most frequently found AFs compared to AFB_2_ (29 %) and AFG_2_ (1 %); no sample was contaminated with AFG_1_. The EC suggested limit for AFB_1_ was exceeded by 24.4 % of tested positive samples (Martinez-Miranda et al., 2019). In Uruguay, maize grains were contaminated by FUMs, DON, and ZEN. The assessment of mycotoxin levels in maize grains indicated that 92 % of samples were contaminated with FB_1_, with a maximum level of 9881 μg/kg, while only 10 % or fewer of the samples exceeded the established EU limit.

In summary (Table 3 and **Table S3**), cereals from the American region (corn, rice, and wheat) were most contaminated by FUMs, particularly FB_3_ (58.05 %), followed by DON (52.91 %). Among the types of AFs, rice (31.32 %) was more affected by AB_1_ than maize (7.28 %). The contamination rate of DON in wheat (98.5 %) samples was higher than in maize (51.61 %) and rice (8.63 %). Rice (23.65 %) samples showed more significant contamination with AFTs than maize (12.95 %) samples. Similarly, the contamination rate of TFU was also significantly observed in maize (85.16 %). Moreover, 50.27 % of rice samples were infected with ZEN, while maize showed a contamination rate of 34.23 %. American region has a vast range of climates, the South American zone having a tropical/ subtropical climate, and suffers a higher risk of contamination by mycotoxins than the North region. The tropical and humid climates in certain areas of South America, particularly Brazil and Colombia, provide optimal conditions for spreading *Aspergillus*, *Alternaria*, *Claviceps*, *Fusarium*, and *Penicillium* species. This favourable environment leads to a significant prevalence of contamination by mycotoxins. Brazil is the most affected country in the American region, particularly in comparison to others, due to its tropical climatic conditions.

**Table 3:** Occurrences of mycotoxins in America (cereals) over the past five years (2019-2024).

**Mycotoxins**	**Matrix**	**Samples/Positive**	**Incidence** **(%)**	**Concentration Range (μg/kg)**	**Sample source**	**Country**	**Year**	**References**
AFTs	Corn grain/silage	291/1828	15.91	n.d-611	Farms, feed production facilities	USA	2021	(Weaver et al., 2021)
	Rice	3/58	5	1.28-2.38	Households	Brazil	2021	(dos Santos et al., 2021)
		68/90	75.6	0.08-19	Local store and markets	Colombia	2019	(Martinez-Miranda et al., 2019)
	Rice	6/53	11	-	Households	Brazil	2020	(Coppa et al., 2020)
AFB_1_	Corn	28/234	11.96	n.d-2.20	Fields	Brazil	2023	(Tonial Simões et al., 2023)
	Rice	7/43	21.21	n.d-47.07	Open box trucks	Mexico	2022	(Molina-Pintor et al., 2022)
	Maize	1/25	4	n.d-21.46	Different sour			
	Corn grain/silage	100/1828	5.47	n.d-606	Farms, feed production facilities	USA	2021	(Weaver et al., 2021)
	Maize flour	25/248	10	1.0-13	Retail stores	Brazil	2020	(Andrade et al., 2020)
	Rice	64/90	71	0.08-19.0	Local store and market	Colombia	2019	(Martinez-Miranda et al., 2019)
AFB_2_	Corn	1/234	0.42	n.d-1.20	Fields	Brazil		(Tonial Simões et al., 2023)
	Rice	26/90	29	0.09-1.42	Local store and market	Colombia		(Martinez-Miranda et al., 2019)
AFG_2_	Rice	1/90	1	0.62-0.62	Local store and market	Colombia		(Martinez-Miranda et al., 2019)
DON	Corn	3/234	1.28	n.d-529	Fields	Brazil	2023	(Tonial Simões et al., 2023)
	Maize grains	59/119	49.41	59–922		Uruguay		(del Palacio et al., 2023)
	Maize grain	87/90	96.66	n.d-20475		USA	2022	(Fusilier et al., 2022)
	Wheat flour	200/200	100	53-2905	Supermarkets	Brazil	2021	(dos Santos et al., 2021)
	Rice	2/32	5.26	13.5-41.0	Fields			(Tralamazza et al., 2021)
	Wheat flour	200/200	100	53-2905	Households			(dos Santos et al., 2021)
	corn grain/silage	1523/1828	83.31	n.d-27000	Farms, feed production facilities	USA	2021	(Weaver et al., 2021)
	Maize flour	89/248	36	3-595	Retail stores	Brazil	2020	(Andrade et al., 2020)
FB_1_	Maize grains	109/119	92	80-9881	Fields	Uruguay	2023	(Del Palacio et al., 2023)
	Corn	107/234	45.72	n.d-4810		Brazil		(Tonial Simões et al., 2023)
	Maize	22/25	88	79.22–16672.62	Fields & Market	Mexico	2022	(Molina-Pintor et al., 2022)
	Maize grain	79/90	88	n.d-45145.82	Fields	USA		(Fusilier et al., 2022)
	Maize	-	87.5	6-6030	Fields	Brazil	2021	(Gasperini et al., 2021)
	Maize flour	234/248	94	21.1-2582	Retail stores	Brazil	2020	(Andrade et al., 2020)
FB_2_	Maize grains	90/119	76	80–4138	Fields	Uruguay	2023	(Calderón et al., 2022)
		76/90	84	n.d- 22,538.63		USA	2022	(Fusilier et al., 2022)
	Maize flour	234/248	94	8-1148	Retail stores	Brazil	2020	(Andrade et al., 2020)
FB_3_	Maize grain	72/90	80	n.d- 17,972.72	Fields	USA	2022	(Fusilier et al., 2022)
TFU	Maize	-	87.5	6-8390	Fields	Brazil	2021	(Gasperini et al., 2021)
	Corn grain/silage	1245/1828	68	n.d-59117	Farms, feed production facilities	USA	2021	(Weaver et al., 2021)
	Rice	26/58	45	3.78-13.95	Households	Brazil		(Dos Santos et al., 2021)
OTA	Flour	5/47	10.6	n.d-1.73	Market	Chile	2022	(Calderón et al., 2022)
ZEN	Corn	4/234	1.7	n.d-428	Fields	Brazil	2023	(Tonial Simões et al., 2023)
	Maize grains	66/119	55.46	-		Uruguay		(del Palacio et al., 2023)
		76/90	84	n.d-4148.75		USA	2022	(Fusilier et al., 2022)
	Rice	39/58	67	0.035-174.28	Households	Brazil	2021	(Dos Santos et al., 2021)
		35/38	92.1	7.5-757.6	Fields			(Tralamazza et al., 2021)
	Maize flour	27de/248	11	24.2-630	Retail stores		2020	(Andrade et al., 2020)

**Note:** AFB_1_, Aflatoxin B_1_; AFB_2_, Aflatoxin B_2_; AFG_1_, Aflatoxin G_1_; AFTs, Aflatoxins total (AFB_1_, AFB_2_, AFG_1_, AFG_2_); DON, Deoxynivalenol; ZEN, Zearalenone; FB_1_, Fumonisins B_1_; FB_2_, Fumonisins B_2_; OTA, Ochratoxin A; FB_1_B_2_, Sum of fumonisins B_1_ & B_2_; TFU, Total fumonisins.

### 4.4 Occurrence of mycotoxins in cereals (corn, rice, and wheat) in Africa

Mycotoxin contamination in cereals and food commodities has been significantly more common in African countries (Table 4). Ghana, located in West Africa, showed 100 % contamination in maize grain samples collected from the storage barns and silos. The maximum concentration in tested positive samples was 945 μg/kg. AFB_1_ (72 %) contamination rate was higher compared to other aflatoxins such as AFB_2_ (50 %), AFG_1_(41 %), and AFG_2_ (15 %). Among all aflatoxins, AFB_1_ concentration was at the highest level of 945 μg/kg (Dadzie et al., 2019). Similarly, in another survey of Ghana conducted by Kortei et al. (2021), the prevalence rate of AFB_1_ was 80 %, ranging from 0.5 to 945 μg/kg. Of 90 samples, 41.25 % exceeded the EC established limit (Kortei et al., 2021).

**Table 4:** Occurrences of mycotoxins in Africa (cereals) over the past five years (2019-2024).

**Mycotoxins**	**Matrix**	**Samples/Positive**	**Incidence** **(%)**	**Concentration Range (μg/kg)**	**Sample source**	**Country**	**Year**	**References**
AFTs	Maize	8/19	42	n.d-252.44	Market	Burkina Faso	2022	(Bandé et al., 2022)
	Rice	9/40	22.5	n.d-4.83				
	Maize	410/800	51.2	n.d-1369	Farmer	Zimbabwe	2021	(Akello et al., 2021)
	Maize dough	53/70	76	1.1-75.9	Household	Togo		(Hanvi et al., 2021)
	Maize	26/100	26	0.080-9.34	Silos & markets	South Africa	2021	(Ekwomadu et al 2021)
	Rice	110/200	55.5	n.d-993	Mills & sellers	Kenya	2021	(Mutiga et al., 2021)
	Maize	35/53	66.9	0.36-3863	Households	Nigeria	2021	(Ezekiel et al., 2021)
	Rice	58/58	100	2.10-248.20	Markets, fields & stores	Nigeria	2020	(Onyedum et al., 2020)
	Corn	20/20	100	270-41.70	Markets, fields & stores	Nigeria	2020	(Onyedum et al., 2020)
	Maize grains	34/34	100	0.5-945	Storage Barns and Silos	Ghana	2019	(Dadzie et al., 2019)
AFB_1_	Wheat	59/136	43.38	17-37.8	Fields	Tunisia	2023	(Aloui et al., 2023)
	Maize flour	12/12	100	1.2-120.5	Public health centre and retail market	Côte d’Ivoire		(N'zi et al., 2023)
	Rice flour	6/6	100	0.1-1.9				
	Maize	72/90	80	0.78-445.01	Market	Ghana	2021	(Kortei et al., 2021)
		23/100	23	0.10-4.96	Silos & markets	South Africa		(Ekwomadu et al 2021)
	Wheat	12/36	33	0.13-49.79	Retailer	Egypt	2020	(Hathout et al 2020)
	Maize grains	21/34	61.776	0.5-821.4	Storage Barns and Silos	Ghana	2019	(Dadzie et al., 2019)
	Rice grain	3/24	12.5	100-200	Market	Egypt		(Moharram et al., 2019)
AFB_2_	Maize	37/100	37	0.009-4.92	Silos & markets	South Africa	2021	(Ekwomadu et al 2021)
	Maize grains	17/34	50	0.5-107.4	Storage Barns and Silos	Ghana	2019	(Dadzie et al., 2019)
AFG_1_	Maize	43/100	43	0.007-1.94	Silos & markets	South Africa	2021	(Ekwomadu et al 2021)
	Maize grains	14/34	41.17	0.5-7.1	Storage Barns and Silos	Ghana	2019	(Dadzie et al., 2019)
	Maize grains	3/19	15.78	0.5-1.0				
DON	Maize	13/30	43	47.6-2055	Market	Algeria	2020	(Mahdjoubi et al., 2020)
		61/123	49.59	8.2-1380	Silos	South Africa		(Ekwomadu et al., 2020)
	Wheat	27/30	90	68.30-1363	Market	Algeria		(Mahdjoubi et al., 2020)
	Corn flour	45/54	83.3	n.d-853	Retail markets	Egypt	2022	(Gab-Allah et al .,2022)
	Wheat flour	28/56	56	n.d-389	Retail markets	Egypt	2022	(Gab-Allah et al .,2022)
FB_1_	Rice flour	5/6	83.3	3.5-82.7	Public health centre and retail market	Côte d’Ivoire	2023	(N'zi et al., 2023)
	Maize flour	12/12	100	50.9-288.6				
	Maize	99/100	99	4.8-1566.7	Silos & markets	South Africa	2021	(Ekwomadu et al 2021)
		29/30	96.6	289-42143	Market	Algeria	2020	(Mahdjoubi et al., 2020)
		121/123	98.37	12.3-8908	Silos	South Africa	2020	(Ekwomadu et al., 2020)
FB_2_	Maize flour	12/12	100	17.2-176.5	Public health centre and retail markets	Côte d’Ivoire	2023	(N'zi et al., 2023)
	Rice flour	6/6	100	1.5-65.7				
	Maize	39/100	39	4.2-239.0	Silos & markets	South Africa	2021	(Ekwomadu et al., 2021)
		27/30	90	27.5-8603	Markets	Algeria	2020	(Mahdjoubi et al., 2020)
		112/123	91.05	7.9-3383	Silos	South Africa		(Ekwomadu et al., 2020)
FB_3_	Maize	98/123	79..67	<7-990	Silos		2020	(Ekwomadu et al., 2020)
FB_1_FB_2_	Maize flour	12/12	100	72.3-465.1	Public health centre and retail markets	Côte d’Ivoire	2023	(N'zi et al., 2023)
	Rice flour	6/6	100	1.8-143.2				
TFU	Maize	700/800	88.9	n.d-40000	Farmers	Zimbabwe	2021	(Akello et al., 2021)
	Maize	75/100	75	4.2-1652.9	Silos & markets	South Africa	2021	(Ekwomadu et al 2021)
OTA	Rice flour	2/6	33.3	0.2-0.3	Public health centre and retail markets	Côte d’Ivoire	2023	(N'zi et al., 2023)
	Maize flour	5/12	41.66	0.1-0.5				
	Maize	95/100	95	1.6-19.44	Silos & markets	South Africa	2021	(Ekwomadu et al 2021)
	Wheat	2/36	5.55	n.d-1.37	Retailer	Egypt	2020	(Hathout et al., 2020)
	Rice grain	3/24	12.5	50-100	Market		2019	(Moharram et al., 2019)
	Wheat/flour	4/100	4	0.6-3.4	Mills	Lebanon		(Elaridi et al., 2019)
ZEN	Maize	53/100	53	0.1-51.30	Silos & markets	South Africa	2021	(Ekwomadu et al 2021)
	Rice	6/30	20	8.6-15.5	Markets	Algeria	2020	(Mahdjoubi et al., 2020)
	Maize	7/30	23.3	20.4-579				
	Maize	41/123	33.33	<0.6-146	Silos	South Africa		(Ekwomadu et al., 2020)
	Wheat	19/30	63.3	9.6-295	Market	Algeria		(Mahdjoubi et al., 2020)

**Note:** AFB_1_, Aflatoxin B_1_; AFB_2_, Aflatoxin B_2_; AFG_1_, Aflatoxin G_1_; AFTs, Aflatoxins total (AFB_1_, AFB_2_, AFG_1_, AFG_2_); DON, Deoxynivalenol; ZEN, Zearalenone; FB_1_, Fumonisins B_1_; FB_2_, Fumonisins B_2_; OTA, Ochratoxin A; FB_1_B_2_, Sum of fumonisins B_1_ & B_2_; TFU, Total fumonisins.

An assessment of mycotoxins in maize samples from North-West South Africa (Silos and market) was conducted. The FB_1_ (99 %), OTA (95 %), TFU (75 %), and ZEN (53 %) were the most frequently found mycotoxins ranging in a concentration of (4.8–1566 μg/kg), (1.6–19.44 μg/kg), (4.2–239.0 μg/kg) and (0.1–51.30 μg/kg) respectively. No maize sample was contaminated with a higher level of OTA/AFTs recommended by the EU (Ekwomadu et al., 2021). The same authors conducted another survey (Ekwomadu et al., 2020) in the same region on maize silos. FB_1_ (98.37 %) was the most prevalent mycotoxin as compared to ZEN (33.3 %) and DON (50 %). The maximum concentration of FB_1_ was reported at 8908 μg/kg level. In Côte d'Ivoire, a West African country N'zi et al. (2023) observed that AFB_1_ and FB_1_B_2_ were in 100 % maize and rice flour samples. While OTA was only detectable in 33.3 % of samples (ranging 0.2–0.3 μg/kg). In Burkina Faso Bandé et al. (2022) confirmed the concentration of AFTs in maize (42 %) and rice (22.5 %) samples with a maximum level of 252.44 and 4.83 μg/kg, respectively. In North Africa, (Aloui et al. (2023) assessed the prevalence level of mycotoxins in wheat samples collected from coastal and continental regions. Of the samples, 43 % were contaminated with AFB1 with a maximum concentration of 37.8 μg/kg, which is higher than the limit set by EC (2.0 μg/kg).

On the African continent **Table S4**, most cereals were contaminated with AFTs (63.19 %) and TFU (60.17 %). Among AFs, AFB_1_ was the most commonly found mycotoxin in wheat, rice, and maize samples (34.48 %), while 48 % of the maize samples were affected with AFB_1_, which was higher than rice (41 %) and wheat (25 %). Among FUMs, FB_1_ was highly reported in maize samples (95.51 %), followed by FB_3_ (87.68 %) and FB_2_ (58.59 %). Furthermore, maize samples were also more vulnerable to TFU (82.97 %) than rice (60.17 %). Likewise, 70.76 % of the wheat Samples in Algeria showed DON contamination at higher concentrations ranging from 68.60 to 1363 μg/kg. The OTA (24.02 %) type of mycotoxins affected the least cereals. In Africa, maize cereals were more affected by mycotoxins than wheat and rice, exposing a significant concern for food safety and public health.

Moreover, geographical and climatic conditions are critical determinants in the mycotoxin contamination of cereals across various global regions. Mycotoxins are significantly influenced by environmental factors that promote the growth of moulds and the subsequent production of mycotoxins. The impact of these elements on contamination levels is apparent throughout Asia, Europe, America, and Africa. Asia endures warm and humid temperatures, especially in monsoon-impacted regions, which promote enhanced levels of FUMs and AFs, particularly in countries including China, India, and Pakistan. In Europe, lower contamination levels of mycotoxins have been observed due to lower temperatures in comparison to tropical regions. However, within Europe, countries located in warmer zones, such as Italy and Spain, are more susceptible to aflatoxins, particularly in corn. Similarly, higher moisture levels during the wheat harvest season are particularly noticeable in Northern Europe concerning ZEN and DON. The study's main finding emphasized that mycotoxins pose a serious health hazard in developing countries.

## 5 Food analytical methods for mycotoxins in cereals

Since their identification, scientists worldwide have been improving methods for detecting and quantifying mycotoxins to be more sensitive, reliable, repeatable, quick, and cheap. The Association of Official Analytical Chemists (AOAC), the European Committee for Standardization (CEN), and the International Organization for Standardization (ISO) are just a few of the many international organizations that have brought together experts to introduce internationally recognized analytical standards. The main aim is to avoid the inconsistencies in results that might occur while using diverse analytical approaches, which could reduce the overall volume of international food trade. The analytical methods should be able to precisely detect the quantities of mycotoxins in foods, although the levels at which they become hazardous are exceedingly low. To achieve satisfactory recoveries, sample collection, homogenization, sample preparation (typically extraction and cleanup), detection, and quantification are the standard procedures for mycotoxin analysis in cereal samples and food commodities (Pereira et al., 2014).

### 5.1 Sampling

Sampling is an essential part of mycotoxin analysis. It determines whether the entire batch of food meets the requirements and assures the accuracy of the results (Pereira et al., 2014; Shephard, 2016). Approximately 80 % of the time dedicated to analysis is allocated to sampling and sample preparation. The uneven distribution of mycotoxins in cereals necessitates precise sampling to ensure that the analyzed sample accurately represents the whole bulk. Various sampling strategies have been designed to ensure consumer safety and safeguard producers (Shephard, 2016). These strategies are implemented by regulatory agencies such as the FDA and the EC, which established Commission Regulation No. 401/2006. This regulation outlines the methodologies for collecting and analyzing mycotoxins in cereal samples, including the specific number and quantity of samples to be acquired (Shephard, 2016). Sampling techniques for processed goods are often simplified since mycotoxins are dispersed less diversely than raw agricultural food samples (Lee & Ryu, 2015).

### 5.2 Choice of solvents to extract mycotoxins in food matrix

The diverse composition of the mycotoxin representative matrices demands various extraction and cleanup operations to prepare a sample for instrumental analysis. In the case of dry matrices, hydration is often necessary to moisten and inflate the samples, facilitating the effective extraction and separation of mycotoxins that are present within the sample. To remove unwanted fatty substances such as lipids and cholesterol, it is crucial to include extra processes to eliminate them from fatty composites. This section provides a study and comparison of widely used extraction solvents (methanol (CH_3_OH), chloroform (CHCl_3_), acetonitrile/methyl cyanide (CH_3_CN/MeCN), acetone (C_3_H_6_O), hexane (C_6_H_14_), and other organic solvents with a specific ratio of organic acid or buffer) and procedures for their usage in mycotoxins analysis. The presence of pigments, essential oils, and fatty acids in the samples poses a challenge in the extraction process, demanding the use of various extraction solvents, such as a combination of ethyl acetate (EtOAc) and formic acid (HCOOH) (di Mavungu et al., 2009).

The choice of solvent depends upon the chemical properties of the desired mycotoxins. Some mycotoxins are soluble only in organic solvents, but others are soluble in water; FUMs are the best example of water-soluble mycotoxins. So, mycotoxins should be extracted from the substrate using the most efficient solvent. Hexane, water, MeCN-water-acetic acid, chloroform, and acetylenediol, alone or at various ratios, have been used for the extraction of mycotoxins (Dadzie et al., 2019; Ishaque Tahir et al., 2021; Ok et al., 2018; Tarazona et al., 2020).

To extract AFs from cereals or cereal-based matrices, CH_3_OH and CH_3_CN combined with H_2_O or CH_3_Cl are considered more effective. Similarly, CH_3_OH and CH_3_CN with a specific ratio of H_2_O are seen as more suitable for extracting OTA. Due to the solubilization and chemical properties of ZEN, a common ratio of CH_3_CN and H_2_O is a well-studied solvent. Additionally, because of the polar nature of DON and its higher solubility in water alone, water is often used; however, in some instances, the combined solvents CH_3_OH/H_2_O and CH_3_CN/H_2_O enhance recovery effectively. Likewise, the combined concentration of CH_3_OH and H_2_O offers a balanced polarity, achieving high recovery and versatility in food matrices based on wheat, maize, and rice. Table 5 represents a compilation of recent studies that report commonly deployed solvents for the extraction of mycotoxins from corn, rice, and wheat, along with the food matrix, sample weight, extraction time, solvent volume, and ratio. Hence, when choosing a solvent system, it is important to consider parameters such as purity, selectivity, recovery, and reactivity (Pereira et al., 2014).

**Table 5:** Examples of the most commonly used solvent for extraction of mycotoxin.

**Mycotoxins**	**Food Matrix**	**Sample portion**	**Solvent volume**	**Solvent ratio**	**Cleaning**	**Country**	**References**
AFB_1_	Maize	25 g	100 mL	CH_3_OH: H_2_O (60:40)	IAC	Serbia	(Udovicki et al., 2021)
	Rice	25 g	100 mL	CH_3_OH: H_2_O (60:40)	IAC	Serbia	(Udovicki et al., 2021)
	Wheat Flour	50 g	50 mL	CH_3_OH: H_2_O (80:20)	IAC	Iran	(Jahanbakhsh et al., 2021)
	Maize	25 g	100 mL	CH_3_CN: H_2_O (90:10)	SPE	Pakistan	(Wajih Ul Hassan et al., 2020)
	Wheat	Wheat	100 mL	CH_3_OH: H_2_O (80:20)	IAC	Lebanon	(Joubrane et al et al., 2020)
	Wheat	50 g	200 mL	CH_3_OH: H_2_O (80:20)	IAC	Egypt	(Hathout et al., 2020)
	Rice	-		CH_3_CN: H_2_O (95:5)	-	China	(Zhao et al., 2019)
	Rice Grain	25 g	100 mL	CH_3_OH: H_2_O (70:30)	IAC	Brazil	(Savi et al., 2018)
AFTs	Corn	5 g	20 mL	CH_3_CN: H_2_O (84:16)	-	Brazil	(Tonial Simões et al., 2023)
	Rice	5 g	-	CH_3_CN: H_2_O (80:20)	QuEChERS	Spain	(Romero-Sánchez et al., 2022)
	Maize	5 g	25 mL	CH_3_OH: H_2_O (70:30)	IAC	Burkina Faso	(Bandé et al., 2022)
	Maize	25 g	100 mL	C_6_H_14_ 100%	IAC	Ghana	(Kortei et al., 2021)
	Maize Dough	25	100	CH_3_OH: H_2_O (80:20)	IAC	Togo	(Hanvi et al., 2021)
	Rice	50 g	175 mL	H_2_O:CH_3_Cl (25:150)	-	Pakistan	(Ishaque Tahir et al., 2021)
	Rice	12.5 g	50 mL	CH_3_OH: H_2_O (80:20)	IAC	Colombia	(Martinez-Miranda et al., 2019)
	Maize grains	2.0 g	4 mL	CH_3_OH:CH_3_CN(60:40)	-	Ghana	(Dadzie et al., 2019)
	Rice	20 g	100 mL	CH_3_OH: H_2_O (80:20)	IAC	Brazil	(Katsurayama et al., 2018)
DON	Maize	25 g	200 mL	100 mL H_2_O	IAC	Turkey	(Golge & Kabak, 2020)
	Wheat	25 g	200 mL	100 mL H_2_O	IAC	Turkey	(Golge & Kabak, 2020)
	Rice	20 g	100 mL	100 mL H_2_O	-	Republic of Korea	(Ok, Lee and Chun, 2018)
	Rice Grain	2 g	8 mL	CH_3_CN: H_2_O: C_2_H_2_O_2_ (80:19.9:0.1)	IAC	Brazil	(Savi et al., 2018)
	Corn	3 g	24 mL	CH_3_OH: H_2_O (70:30)	-	Brazil	(Tonial Simões et al., 2023)
	Maize	2 g	8 mL	CH_3_CN: H_2_O:C_2_H_2_O_2_ (80:19:1)	-	Spain	(Tarazona et al., 2020)
OTA	Wheat	Wheat	100 mL	CH_3_OH: H_2_O (80:20)	IAC	Lebanon	(Joubrane et al., 2020)
	Wheat	50 g	100 mL	CH_3_CN: H_2_O (60:40)	IAC	Egypt	(Hathout et al., 2020)
FB_1_	Grains	5 g	20 mL	CH_3_CN: H_2_O: C_2_H_2_O_2_ (79:20.9:1)	-	Croatia	(Kifer et al., 2021)
	Raw Maize	1 g	10 mL	CH_3_CN: H_2_O:C_2_H_2_O_2_ (79:20.9:1)	-	China	(Hu et al., 2019)
FB_1_FB_2_	Corn	3 g	15 mL	CH_3_CN: H_2_O (1:1)	-	Brazil	(Tonial Simões et al., 2023)
OTA	Corn	3 g	12 mL	CH_3_CN: H_2_O: CH₃COOH (700:290:10)	-	Brazil	(Tonial Simões et al., 2023)
	Flour	5 g	20 mL	CH_3_OH: H_2_O (80:20)	IAC	Chile	(Calderón et al., 2022)
ZEN	Maize	25 g	125 mL	CH_3_CN: H_2_O (75:25)	IAC	Turkey	(Golge & Kabak, 2020)
	Wheat	25 g	125 mL	CH_3_CN: H_2_O (75:25)	IAC	Turkey	(Golge & Kabak, 2020)
	Corn	3 g	24 mL	CH_3_OH: H_2_O (70:30)	-	Brazil	(Tonial Simões et al., 2023)
	Rice Grain	2 g	8 mL	CH_3_CN: H_2_O: C_2_H_2_O_2_ (80:19.9:0.1)	IAC	Brazil	(Savi et al., 2018)

**Note:** AFTs; Aflatoxins total, AFB_1_; Aflatoxin B_1_, AFB_2_; Aflatoxin B_2_, AFG_2_; Aflatoxin G_2_, DON; Deoxynivalenol, ZEN; Zearalenone, FB_1_; Fumonisins B_1_, FB_2_; Fumonisins B_2_, OTA; Ochratoxin A, FB_1_B_2_; Sum of fumonisins B_1_ & B_2_.

### 5.3 Extraction techniques for mycotoxins analysis

Before adopting methods to perform extraction and cleanup, it is important to examine three primary factors: a) the chemical characteristics of the mycotoxins, b) the nature of the food matrix, and c) the final detection method that will be utilized. The most commonly used extraction techniques for analyzing mycotoxins include solid-liquid extraction (SLE), liquid-liquid extraction (LLE), quick, easy, cheap, effective, rugged, and safe (QuEChERS), pressurized liquid extraction (PLE)/accelerated solvent extraction (ASE), supercritical fluid extraction (SFE), microwave-assisted extraction (MAE), and vortex-assisted liquid-liquid dispersive microextraction (VADS-ME).

Despite its origins in mycotoxin analysis, the QuEChERS approach has evolved to allow the simultaneous detection of several mycotoxin groups in an extensive range of food matrices (Pereira et al., 2014). The process begins with a MeCN-water extraction, and inorganic salts are added to promote liquid-liquid partitioning. Consequently, mycotoxins are subjected to the organic phase, whereas some polar matrix components are retained in the aqueous layer. Subsequently, a dispersive solid phase extraction (DSPE) is utilized to diminish the existence of additional matrix components in the organic phase (González-Jartín et al., 2019). The QuEChERS method has analyzed several mycotoxins in various food matrixes, including OTA, ZEN, DON, FUMs, and AFs (Desmarchelier et al., 2014; Do et al., 2020; Zhao et al., 2019). This extraction method utilizes a limited amount of MeCN as an organic solvent, proving economically viable, rapid, and independent of a specialized workforce.

LLE relies on the varying solubility of toxins in the aqueous phase and the immiscible organic phase. The molecule is separated from the matrix by extracting it into one solvent, while the remaining matrix is left in the other solvent (Turner et al., 2009). It is not frequently used for mycotoxin extraction from cereals such as corn, wheat, and rice but is commonly used for liquid samples. SLE is a facile technique for extracting mycotoxins from solid food matrices with different consistencies. The extraction process involves weighing a homogeneous sample and adding an extraction solvent. The mixture is then agitated using a shaker, mostly incorporating ultrasonic extraction, homogenization, and shaking (Xie et al., 2016; L. Zhang et al., 2018). It has been verified that this technique may be employed to remove diverse mycotoxins from cereals and cereals-based products.

Solvent extraction techniques, including SFE, MAE, and ASE, have been utilized in recent research. Despite the potential expense, these approaches surpass SLE in terms of the effectiveness of extraction, need less chemical solvent, and are quicker (Santos et al., 2022). These techniques could prove somewhat more costly than SLE, nevertheless, they are quicker, use a smaller quantity of solvent, and have superior extraction efficiency. Sample centrifugation and filtration are carried out prior to additional cleanup procedures to remove any potential particles that might interfere (Alshannaq & Yu, 2017). A comparison of the most frequently used extraction techniques for analyzing mycotoxins is summarised in Table 6.

**Table 6:** Extraction methods to analyze mycotoxins, advantages and disadvantages.

**Extraction techniques**	**Advantages**	**Disadvantages**	**References**
SLE	High recoveries, smaller solvents volume needed.	Matrix effect requires additional steps for cleanup.	(Leite et al., 2020;Soriano del Castillo, 2009;Turner et al., 2009)
QuEChERS	Rapid, facile and versatile method, cost-effective, good reproducibility and accuracy, detection of lower level i.e. μg/L.	Not suitable for lipophobic compounds due to demanding additional enrichment steps.	(Al-Jaal et al., 2019;González-Curbelo et al., 2015;Perestrelo et al., 2019;Yang et al., 2020)
LLE	Efficient for small-scale arrangements.	Potential sample loss, takes long times.	(Leite et al 2020;Miklós et al., 2020;Song et al., 2013)
PLE	Good extraction, required small solvents volume, rapid, automated, and time-saving.	Matrix effect, not economic.	(Alvarez-Rivera, Bueno, Ballesteros-Vivas, Mendiola and Ibañez, 2020, Xie, Chen and Ying, 2016, Zhang, Dou, Zhang, Logrieco and Yang, 2018)
SFE	Rapid, requires smaller solvent volume, beneficial for thermally labile compounds.	Expensive, demanding more specialized equipment, limited solvating power, matrix dependence.	(Miklós et al., 2020;Turner et al., 2009)
MAE	Rapid required a smaller volume of solvents as compared to conventional extraction, simultaneously extraction, cheaper as compared to SFE and PLE.	Thermal degradation, costly, scale-up challenges, matrix effect, extraction solvent must have the ability to absorb microwave, patience till the vessel cools down.	(Sparr Eskilsson & Björklund, 2000)
VALDS-ME	Quick, good efficiency, low-density solvents.	Demanding optimized parameters.	(Somsubsin et al., 2018)

**Note:** SLE, solid-liquid extraction; LLE, liquid-liquid extraction; QuEChERS, Quick, Easy, Cheap, Effective, Rugged, and Safe; PLE/ASE, pressurized liquid extraction/ accelerated solvent extraction; SFE, supercritical fluid extraction; MAE, microwave-assisted extraction; VADS-ME, Vortex-assisted liquid-liquid dispersive microextraction.

The comparison investigation shows that the QuEChERS method is the more effective extraction approach for mycotoxins in cereal samples with varying polarities. Its speed, adaptability, cost-effectiveness, and ability to detect trace amounts of mycotoxins make it particularly advantageous for high-throughput applications. However, it is essential to acknowledge its limitations regarding lipophobic substances, which may require additional enrichment techniques. The choice of an extraction process should be guided by the specific mycotoxin being studied, the characteristics of the grain matrix, and the analytical requirements. QuEChERS is supported for its efficiency, accuracy, and affordability while recognizing that other methods may be necessary to address its limitations. Additionally, optimizing the solvent and adsorbent used in each research study is crucial for accurate measurements with the QuEChERS technique.

### 5.4 Enrichment of mycotoxins extract from food matrix

Effectively cleaning the extract is necessary to mitigate matrix effects and remove contaminants that may disrupt the subsequent identification of mycotoxins. The process of purifying the extract enhances specificity and sensitivity, enhancing the accuracy and precision of quantification. Solid phase extraction (SPE) and immunoaffinity columns (IAC) are widely employed techniques for cleaning mycotoxins. Both of these methods are preferred due to their speed, effectiveness, reproducible results, and ability to selectively target a broad spectrum of mycotoxins (González-Curbelo et al., 2015; Razzazi-Fazeli & Reiter, 2011).

The SPE method utilizes solid absorbents, often contained in cartridges, to absorb mycotoxins. These cartridges are cleaned to eliminate impurities and retain the mycotoxins (Huertas-Pérez et al., 2017). SPE is characterized by its speed, efficiency, and capacity to produce consistent results. However, it has certain limitations, such as being unable to utilize a single cartridge to detect all types of mycotoxins. Furthermore, the efficiency can be influenced by other factors, including the nature of the solvent, as well as the pH and ionic strength of the sample (Wang et al., 2016). Commercially, adsorbents such as octadecylsilyl (C18), hydrophilic-lipophilic balance (HLB), amino-propyl, and silica gel can be utilized for SPE. However, most commercially available cartridges are unsuitable for high-throughput screening of multiple-class mycotoxins (Jiang et al., 2018; Wang et al., 2016). Carbon nanomaterials and magnetic nanoparticles (MNPs) have recently been utilized as substitute sorbents due to their high absorption capabilities. Multiwall carbon nanotubes (MWCNTs) effectively detect type A trichothecenes in rice, maize, and wheat (Dong et al., 2015). Additionally, MWCNT-MNPs were utilized as sorbents to purify ZEN (Han et al., 2017) in maize and type A trichothecenes in coix (Dong et al., 2016).

**Fig. 6:**
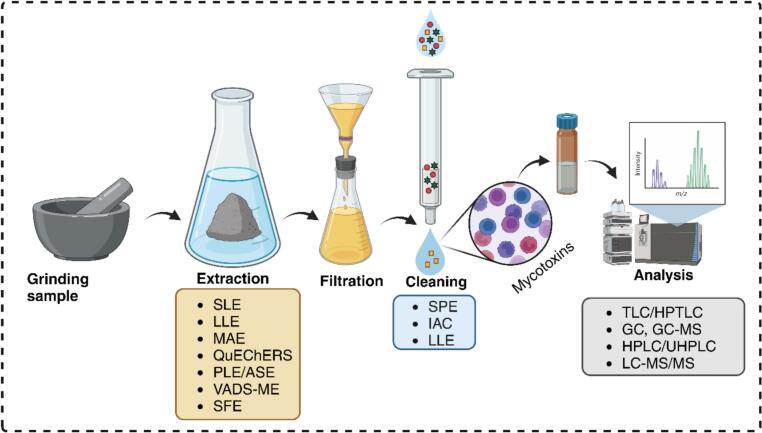
Flowsheet diagram for analyzing mycotoxins in cereals or cereals-based products (image redrawn from https://www.biorender.com). Note: SLE, solid-liquid extraction; LLE, liquid-liquid extraction; MAE, microwave-assisted extraction; QuEChERS, Quick, Easy, Cheap, Effective, Rugged, and Safe; PLE, pressurized liquid extraction; ASE, accelerated solvent extraction; VADS-ME, Vortex-assisted liquid-liquid dispersive microextraction; SPE, solid phase extraction; IAC, immunoaffinity column; SFE, supercritical fluid extraction.

Immunoaffinity columns (IAC) comprise a solid phase support that has been activated and is attached to a specific antibody. As the sample extract flows down the column, mycotoxins specifically attach to the column antibodies, while impurities and other constituents in the sample are eliminated during a subsequent washing phase. Subsequently, the mycotoxin is extracted using a miscible solvent, such as CH_3_OH, effectively separating them from the column (Singh & Mehta, 2020). This approach has excellent selectivity compared to C8, C18, and SiO_2_ but has numerous drawbacks: its expensive cost, restricted column use, and capability to isolate only a certain type of mycotoxins or a cluster of structurally similar mycotoxins (Ibrahim, 2020). In addition, there is a potential danger of antibody denaturation when exposed to certain chemical solvents, as well as the chance of cross-reactivity and the formation of non-specific interactions (Singh & Mehta, 2020). IAC is often used to extract the most prevalent mycotoxins, including AFs, ZEN, OTA, FUMs, and DON, from wheat maize and rice (Bandé et al., 2022; Calderón et al., 2022; Golge & Kabak, 2020; Jahanbakhsh et al., 2021; Kortei et al., 2021; Udovicki et al., 2021). Certain columns also enable simultaneous extraction of several mycotoxin classes (Singh & Mehta, 2020). To analyze more intricate materials, it is occasionally necessary to combine IAC with other extraction procedures, such as SPE (Şenyuva & Gilbert, 2010). After cleaning, the combined extract is processed to remove any remaining moisture. Solvents are evaporated, preferably in an (inert environment) N_2_ stream that can concentrate the extracted solvent. Last, the residues are reconstituted in clean organic solvents C_2_H_3_N or CH_3_OH and then employed in calculations. Fig. 6 shows the flowsheet diagram for analyzing mycotoxins in cereals or cereals-based products.

## 6 Chromatographic analysis of mycotoxins in cereals

In order to comply with the maximum allowable limits (MLs) set by various countries, researchers must employ techniques that may provide precise and accurate results in the analysis of mycotoxins (Pereira et al., 2014). The prevalence of mycotoxins in feed and food products has been studied using various approaches since their discovery. Official analytical techniques are provided in the AOAC for analyzing mycotoxins in food and feedstuffs (Rai et al., 2018). Because of their diversity, numerous approaches have been established to simultaneously detect many mycotoxins due to their presence in the same matrix (El-Sayed et al., 2022). The three main categories of mycotoxins detection techniques are immunochemical, spectroscopic, and chromatographic. Enzyme-linked immunosorbent assay (ELISA) methods can identify the vast range of mycotoxins and are widely used for screening. However, the main drawbacks are the matrix effect, the requirements of full validation, and the number of false +Ve (due to cross-reactivity and matrix dependence) or false -Ve (because of low sensitivity) (K. Zhang & Banerjee, 2020). The speed, low cost, and lack of destruction of spectrometry have led to its implementation in various situations. However, the required regulatory precision is not provided by infrared spectroscopic methods for detecting and quantifying mycotoxins. These quantization models are excessively imprecise because of the sensitivity limits of the devices used, requiring extensive sample preparation and a large number of highly skilled workforce (Levasseur-Garcia, 2018).

Chromatography is the principal analytical technique for analyzing mycotoxins in food and feed samples (Shephard, 2016). In comparison to other methods, chromatographic approaches enable a broad spectrum of analytes in addition to superior analytical reliability and robustness (Vargas et al., 2021). These procedures rely on partitioning the chemical mixture into its constituent components by distributing them between two phases, i.e., the mobile and stationary phases (Shephard, 2016). Thin-layer chromatography (TLC), high-performance thin-layer chromatography (HPTLC), high-performance liquid chromatography (HPLC), ultra-high-performance liquid chromatography (UHPLC), liquid chromatography-tandem mass spectrometry (LC-MS), and gas chromatography (GC) have been employed in the identification and quantification of mycotoxins from cereals and cereal-based products. A brief comparison of different chromatographic techniques used to analyze mycotoxins is provided in Table 7.

**Table 7:** Pros and cons of chromatographic techniques used to detect mycotoxins.

**Techniques**	**Pros**	**Cons**	**References**
TLC	A cost-effective, user-friendly, rapid screening approach, offering semi-quantitative mycotoxins analysis, detecting multiple mycotoxins, improving precision and accuracy is accomplished via the advancement of HPTLC.	Constrained resolution, identifying capabilities are confined to non-specific methodologies, compatibility challenges (unsuitable for highly automated techniques), not sensitive enough to be deemed accurate, Variation in spotting sample, temperature and humidity may affect screening, degradation of specific mycotoxins in excess UV.	(Lin et al., 1998;Levasseur-Garcia, 2018;Vargas Medina et al., 2021;Lillard and Lantin, 1970;Carnaghan et al., 1963;Zhang & Banerjee, 2020)
HPLC	Rapid separation, good precision and accuracy as compared to TLC, efficient sensitivity/recovery, and ease of use.	To minimize the impact of signal quenching, extensive cleanup/pre-column/post-column derivatization step is required.	(Alshannaq and Yu, 2017;Chandravarnan et al., 2022;Zhang & Banerjee, 2020)
LC-MS	Simultaneous determination potential of multiple mycotoxins, selective/sensitive screening, an additional feature of structural (molecular) information, detection limit low, unmatchable high resolution, minimal sample pretreatment, more advanced, accurate mass libraries.	Demanding additional steps for extraction and cleanup is expensive.	(Chandravarnan et al., 2022;Shanakhat et al., 2018;Malachová et al., 2018;Vargas et al., 2021;Zhang et al., 2020)
GC	Superior separation, coupled with ECD, FID, MS, and tandem MS.	Due to the high polarity and non-volatile nature of mycotoxins, an additional derivatization step is required, which may cause sample degradation, drift response, valid for thermal stable and volatile mycotoxins, column blockage, non-linearity, and risk of contamination.	(Singh and Mehta, 2020;Janik et al., 2021;Chandravarnan et al., 2022)

**Note:** ECD; Electron capture detector, FID; flame ionization detector.

### 6.1 Thin layer (TLC) and high-performance thin layer chromatography (HPTLC)

TLC is one of the oldest chromatographic techniques that offer the cost-effective detection of various mycotoxins from cereal and cereals-based products (Shephard, 2016; J. Yang et al., 2014). TLC consists of a stationary phase comprised of alumina, silica/cellulose, and immobilized on an inert substance such as plastic or glass that serves as a matrix. The mobile phase comprises various mixtures of methanol, acetonitrile, and water, transporting the sample within the solid stationary phase (Wacoo et al., 2014). The simplicity and bright spots under UV light make it a crucial tool for evaluating various mycotoxins. This technique was intended for qualitative as well as quantitative mycotoxin analysis (Andrade et al., 2013; Caldas & Silva, 2007; Rizzo et al., 2004). TLC has been deployed to detect AFTs in different rice and wheat samples from Pakistan (Ishaque Tahir et al., 2021; Zahra et al., 2019). Pradhan and Ananthanarayan (2020) employed high performance thin layer (HPTLC, another updated version of TLC) to effectively assess the level of AFB_1_ contamination in several cereal samples obtained from the Mumbai market.

Some difficulties may also arise during the TLC procedure's spotting, TLC plate production, and interpretation phases. This low-tech, low-cost technique can only be used for qualitative reasons, as its detection limits in some cases are too low to be helpful. Moreover, TLC is incompatible with the automated system and is concerned about sample destruction during preparation (Levasseur-Garcia, 2018; Vargas et al., 2021). In comparison to TLC, HPTLC allows more selective and accurate quantitative measurements. The primary distinguishing features between TLC and HPTLC are the particle size of the stationary phases, sensitivity, and data processing method (Gurav & Medhe, 2018). Therefore, current research has emphasized utilizing techniques that identify and measure several mycotoxins with exceptional specificity and sensitivity, resulting in more precise outcomes.

### 6.2 High/ultra high-performance liquid chromatography (HPLC/UHPLC)

One of the most used separation approaches, high-performance liquid chromatography (HPLC), has many applications in different fields, including medicine, the environment, food science, and diagnosis. This method can analyze mycotoxins when coupled with ultraviolet detection and differential absorbance detection/ fluorescence detection. Typical detectors utilized in HPLC mycotoxin analysis include fluorescence (FLD), UV–visible (UV), and photodiode array (PDA) (Valenta, 1998). HPLC-FLD is the most often employed technique for measuring the content of AFs, OTA, ZEN, and FUMs (J. Liu et al., 2016). This approach has several advantages, notably high accuracy and sensitivity and the ability to analyze samples in a single analysis. An HPLC-UV was used to validate and standardize the QuEChERS extraction clean-up to evaluate DON from wheat samples. The LOD and LOQ of DON from wheat samples were 16.7 and 55.5 μg/kg (Rosa Seus Arraché et al., 2018).

Certain mycotoxins, such as AFs and OTA, have characteristic fluorescence and can be directly identified by HPLC-FLD. Derivatization is required for mycotoxins such as FB_1_ with non-existent chromophores in their structure (Miklós et al., 2020; L. Zhang et al., 2018). For instance, (Pokrzywa & Surma, 2022) utilized HPLC-FLD to determine the contamination level of ZEN and FB_1_B_2_ in maize and wheat flour. The limit of detection (LOD) and limit of quantification (LOQ) were observed in the range of (4.5–75 μg/kg) and (15–150 μg/kg) respectively. In another study, HPLC-FLD was deployed to determine the concentration of OTA in wheat flour, corn flour, and wheat grain samples with LOD of 0.6 μg/kg and LOQ of 1.2 μg/kg (Hajok et al., 2019). An HPLC approach employing fluorescence detection was used to examine 246 samples for AFTs (HPLC-FLD). In another study, AFB_1_, AFB_2_, AFG_1_ AFG_2_, and OTA in maize samples were assessed using HPLC-FLD, and the LOD of and LOQ were determined to be 0.5, 0.1, 0.05, 0.1, 0.01 μg/kg and 0.5, 0.1, 0.05, 0.3, and 0.03 μg/kg respectively (Wajih Ul Hassan et al., 2020). HPLC equipped with FLD has been reported in different studies to assess the level of AFTs, OTA, and ZEN in cereal and cereals-derived products (Calderón et al., 2022; Dadzie et al., 2019; Hanvi et al., 2021; Hathout et al., 2020; Jahanbakhsh et al., 2021).

DAD detectors acquire spectrum profiles from molecular mixtures or chromatographically isolated materials. An HPLC-DAD detector is coupled with a separation system that allows elution based on molecular weight, hydrophobicity (reverse phase), or ionic load. This feature makes them significant for HPLC applications (Singh and Mehta, 2020). Hence, mycotoxins can be quantified using HPLC, a simple, repeatable, and generally accepted approach that is also being measured using a modified HPLC procedure coupled with mass spectrometry. Table 8 provides a concise summary of the various detection chromatographic techniques that are being utilized to analyze mycotoxins in food matrix with LODs and LOQs. Jorgenson invented ultra-high-pressure liquid chromatography (UHPLC) in 1997. It is based on the difference in the particle size (1.3–2.5 μm) of the packed column and operating pressure (1250 bar). Since its commercial availability in 2004, UHPLC has garnered considerable attention because it significantly boosts the throughput of conventional HPLC systems (Fekete et al., 2014).

**Table 8:** Most commonly, chromatographic techniques used for analysis of mycotoxins in cereals.

**Techniques**	**Mycotoxins**	**Food Matrix**	**LOD (μg/kg)**	**LOQ (μg/kg)**	**Country**	**Year**	**References**
HPLC-DAD	DON	Wheat flour	50	100	Poland	2022	(Pokrzywa & Surma, 2022)
		Maize flour					
HPLC-FLD	ZEN	Wheat flour	4.5	15			
		Maize flour					
HPLC-FLD	FB_1_B_2_	Maize flour	75	150			
HPLC-FLD	OTA	Wheat flour	0.6	1.2	Poland	2019	(Hajok et al., 2019)
		Corn flour					
		Wheat grain					
HPLC-FLD	AFB_1_	Rice	0.014	0.046	Colombia	2019	(Martinez-Miranda et al., 2019)
	AFB_2_		0.004	0.014			
	AFG_1_		0.014	0.046			
	AFG_2_		0.004	0.014			
HPLC-FLD	AFB_1_	Wheat	0.0042	0.027	Lebanon	2020	(Joubrane et al., 2020)
	OTA		0.0034	0.015			
HPLC-FLD	AFB_1_	Wheat flour	0.003	0.01	Iran	2021	(Jahanbakhsh et al., 2021)
HPLC-FLD	AFs	Maize	0.99	2.97	Ghana	2021	(Kortei et al., 2021)
HPLC-FLD	AFB_1_	Wheat	0.04	0.1	Egypt	2020	(Hathout et al., 2020)
	OTA	Wheat	0.02	0.07			
HPLC-FLD	AFs	Maize dough	0.08	0.15	Togo	2021	(Hanvi et al., 2021)
HPLC-FLD	AFs	Maize	0.5	0.1	Ghana	2019	(Dadzie et al., 2019)
HPLC-FLD	AFB_1_	Maize	0.5	0.15	Pakistan	2020	(Wajih Ul Hassan et al., 2020)
	AFB_2_	Maize	0.1	0.3			
	AFG_1_	Maize	0.05	0.15			
	AFG_2_	Maize	0.1	0.3			
	OTA	Maize	0.01	0.03			
	AFB_2_	Rice	0.012	0.039			
	AFG_1_	Rice	0.011	0.038			
	AFG_2_	Rice	0.004	0.012			
HPLC-FLD	ZEN	Maize	2.5	8.5	China	2022	(Tan et al., 2022)
HPLC-FLD	OTA	Flour	0.3	1	Chile	2022	(Calderón et al., 2022)
HPLC-PDA	DON	Maize	14.08	46.9	Turkey	2020	(Golge & Kabak, 2020)
	DON	Wheat	21.7	72.3			
HPLC-FLD	ZEN	Maize	1.06	3.5			
	ZEN	Wheat	1.12	3.7			
HPLC-UV	DON	White rice	6.4	21.3	Republic of Korea	2018	(Ok et al., 2018)
		Brown rice	8.4	27.9			
		Bran	10	33.5			
LC-MS/MS	AFB_1_	Rice	0.16	0.54	Spain	2022	(Romero-Sánchez et al., 2022)
LC/MS-MS	FB_1_	Grains	2.4	8	Croatia	2021	(Kifer et al., 2021)
LC/MS-MS	FB_1_	Raw maize	7	28	China	2019	(Hu et al., 2019)
LC/MS-MS	FB_1_	Maize	2.4	8	South Africa	2020	(Ekwomadu et al., 2020)
LC/MS-MS	DON	Maize	15	50	Albania	2021	(Topi, Babič et al., 2021)
	FB_1_	Maize					
	DON	Wheat					
LC-MS/MS	AFTs	Maize	10	25	Italy	2020	(Bertuzzi et al., 2020)
	DON	Wheat	10	25			
LC-MS/MS	AFs	Maize	0.16	0.54	Morocco	2019	(Ouakhssase, Chahid et al., 2019)
LC/MS-MS	AFB_1_	Wheat	0.1208	0.2608	Ethiopia	2022	(Fikadu et al., 2022)
	AFB_2_	Wheat	0.0302	0.0738			
	AFG_1_	Wheat	0.0328	0.079			
	AFG_2_	Wheat	0.1272	0.3232			
LC/MS-MS	AFB_1_	Rice	0.03	0.5	China	2019	(Zhao et al., 2019)
LC/MS-MS	AFB_1_	Maize	0.28	0.5	Serbia	2021	(Udovicki et al., 2021)
		Rice	0.17	0.5			
UHPLC-FLD	AFTs	Maize	0.8	2.9	Burkina Faso	2022	(Bandé et al., 2022)
UHPLC-MS-MS	FB_1_	Maize	0.02	0.06	China	2022	(Wang et al., 2022)
		Wheat	0.02	0.06			
UHPLC-MS/MS	DON	Maize	11.9	36	Spain	2020	(Tarazona et al., 2020)
UHPLC-MS/MS	AFB_1_	Rice	0.06	0.2	China	2022	(Hu et al., 2022)
HPTLC	AFB_1_	Cereals	-	-	India	2020	(Pradhan & Ananthanarayan, 2020)
TLC	AFTs	Rice	-	-	Pakistan	2021	(Ishaque Tahir et al., 2021)

**Note:** AFTs, Aflatoxins total; AFB_1_, Aflatoxin B_1_; AFB_2_, Aflatoxin B_2_; AFG_2_, Aflatoxin G_2_; DON, Deoxynivalenol; ZEN, Zearalenone; FB_1_, Fumonisins B_1_; FB_2_, Fumonisins B_2_; OTA, Ochratoxin A; FB_1_B_2_, Sum of fumonisins B_1_ & B_2_; TFU, Total fumonisins.

### 6.3 Liquid chromatography-tandem mass spectrometry (LC-MS/MS)

The mass-to-charge (*m*/*z*) ratio of product ions following precursor ion selection and fragmentation is the core concept of MS/MS (Pascale et al., 2019). In mycotoxin analysis, LC-MS has superior selectivity, analyte/matrix scope, and identification. Advances in LC-MS mycotoxin analysis are yielding positive results. Previously, tailored research targeted a few analytes. Modern methods can identify mycotoxins using multi-mycotoxin methods. A strong balance between sample preparation, chromatographic separation, and MS detection allows multi-detection techniques to identify many mycotoxins in tiny quantities. Using tandem and high-resolution techniques, LC-MS can investigate mycotoxins in targeted, post-targeted, and untargeted modes (Malachová et al., 2018). Recent research has utilized advanced LC-MS setups to analyze multiple mycotoxins.

The two most prevalent ionization methods employed in developing single- and multi-class mycotoxin LC-MS techniques are electrospray ionization (ESI) and atmospheric pressure chemical ionization (APCI). These methods demonstrated effective sensitivity for most mycotoxins in multi-trace approaches. Nonetheless, specific mycotoxins like A and B-trichothecenes yielded improved results with APCI interfaces (Aramendía et al., 2010; Berthiller et al., 2005). Higher mean recoveries, ranging from 90 to 117 % for maize and 87–112 % for wheat, were reported using an LC-MS setup with integrated MRM in ESI+ (3.4) and ESI- (30) modes. The LOD and LOQ for both were 15.0 μg/kg and 50.0 μg/kg (Topi et al., 2021). Similarly, the LOD and LOQ for rice samples were 0.17 and 0.5 μg/Kg, while for maize, they were 0.28 and 0.5 μg/Kg (Udovicki et al., 2021).

To analyze mycotoxins various instruments, such as triple-quadrupole (QqQ), ion trap, and time-of-flight (TOF), are employed for both targeted and untargeted detection. Hybrid instruments combine analyzers, such as the quadrupole orbital ion trap (QeOrbitrap), enhancing detection and effectiveness. Tandem mass spectrometry (MS/MS) with triple QqQ and quadruple linear ion trap (QLIT) is the cost-effective method for precise quantification. Although these analyzers have theoretical resolution, QqQ and QLIT in multiple reaction monitoring (MRM) mode offer selectivity and sensitivity. Identifying mycotoxins in MS/MS requires recording a precursor ion and at least two product ions, as done in other pollutant studies (Vargas Medina et al., 2021). AFTs showed recoveries of 50–120 % below 1 μg/Kg using the QuEChERS technique and 70–110 % for 1–10 μg/Kg levels, except AFB_2_ (Ouakhssase et al., 2019). The LC system coupled to QqQ with ESI^+^ mode assessed AFs contamination in wheat, with LODs for AFB_1_, AFB_2_, AFG_1_, and AFG_2_ at 0.120, 0.302, 0.328, and 0.1272 μg/Kg, respectively. Recovered spiked samples averaged 70.80 %–77.23 % (Fikadu et al., 2022). The QqQ and an ESI interface yielded excellent recoveries of AFB_1_ in rice within the range of 88.5 % to 103.7 %, with precisions below 20 % (Zhao et al., 2019). The LC-MS had a QqQ (ESI^+^) interface, and MRM was used for sensitivity. The LOD and LOQ for rice samples were 0.17 and 0.5 μg/Kg, while for maize, they were 0.28 and 0.5 μg/Kg (Udovicki et al., 2021). Likewise, the LOD and LOQ for AFB_1_, AFB_2_, AFG_1_, and AFG_2_ in maize and wheat samples ranged from 0.01 to 0.02 μg/Kg to 0.04–0.06 μg/Kg, respectively were reported (Wang et al., 2022). Liang et al. (2024) designed UHPLC-MS/MS to evaluate five mycotoxins in rice and noodles, studying LODs and LOQs of AFB_1_, AFB_2_, AFG_1_, and AFG_2_ at 0.06, 0.06, 0.06, 0.12 and 0.2, 0.2, 0.2, 0.4 μg/Kg respectively.

### 6.4 Gas chromatography

Like other chromatographic techniques, GC analysis uses a liquid/gas partition to determine analyte concentrations. The study involves the differential partitioning of analytes between liquid and gas phases. A carrier gas converts the test specimen to a gas, evaporates it, and transports it to the stationary phase. There is a balanced distribution of the different chemical components of the sample between the two phases, stationary and mobile. In practice, the flow rate through the column will be determined by the partition coefficient for each analyte component. After separating volatile chemicals, they are identified using a universal GC detector, such as a Flame Ionization Detector (FID) or an Electron Capture Detector (ECD). GC is not commonly employed because of its high price and the necessity for intensive pre-analysis cleaning (Al-Bukhaiti et al., 2017).

## 7 Conclusion and future perspectives

The coupling of high-resolution mass spectrometry (HRMS) with artificial intelligence-based data processing is expected to substantially improve the sensitivity and precision of mycotoxin detection. Advanced methodologies such as ambient ionization mass spectrometry and portable biosensors may facilitate prompt, on-site determinations of mycotoxins in cereal or cereals-based products. Future research ought to emphasize on formulating eco-friendly extraction solvents, including deep eutectic solvents (DES) and ionic liquids, to diminish reliance on traditional organic solvents while preserving high extraction efficiency. Moreover, microwave-assisted and ultrasound-assisted extraction techniques demonstrate the potential for enhancing both efficiency and yield in the extraction of mycotoxins. Progress in biological control methods, including the application of probiotic bacteria, enzymatic degradation, and nanotechnology-based adsorbents, could result in more efficient procedures for mycotoxin elimination. Moreover, examining the genetic alteration of crops to improve resistance to fungal contamination could be essential for lowering mycotoxin levels. In addition, limited data support significant challenges regarding the variety, origin, binding mechanism, transformation, control measures, and detection of matrix-associated mycotoxins. Future studies should also focus on the extent of the issue concerning matrix-associated mycotoxins in cereals of cereals-based products.

Most countries have already implemented restrictions to limit exposure to protect their citizens from the health concerns associated with mycotoxins. Access to more data can explore emerging measurement technologies and enhance understanding of regulatory guidelines. There needs to be an increased emphasis on international collaboration and data sharing to monitor global trends in mycotoxins and implement effective regulatory measures to ensure food safety. This entails establishing standardized protocols for mycotoxin analysis and surveillance programs to track contamination levels in staple foods across different regions. In regions where raw materials with distinct temperatures and trophic levels are produced, it becomes particularly important to have effective livestock feed free of mycotoxins. Predicting the threat of contamination on a regional and area-specific level requires an exhaustive pre- and post-harvest support system, especially an online technical expert system. Looking to the future, the focus should be on genomics and proteomics approaches to develop resistance to moulds. Overall, the prospects for addressing the challenges posed by food mycotoxins are promising, as evidenced by ongoing research. This effort will drive progress in the field and help mitigate the risks to human health and economic stability.

In conclusion, mycotoxins in cereals significantly threaten human health, nutritional value, and economic worth, creating a serious issue for the food industry. After a thorough evaluation, the five most prevalent mycotoxins in cereals globally were AFs, OTA, DON, FUM, and ZEN, primarily linked to fungal species such as *Aspergillus* and *Fusarium*. Various environmental, biological, and logistical factors exacerbate this contamination. Mycotoxin levels in various cereals from the African region continue to be above recommended standards, according to data collected from 2019 to 2024, followed by the Asian, American, and European regions. This is because Africa is particularly prone to the development of moulds due to its diverse topography, climatically changing landscape, and agnomical practices. Recent innovations have allowed for the simultaneous detection of several targets, accomplished through enormous composite cleaning stages like QuEChERS, moving beyond the detection of single compound determination. IAC cleaning combined with LC-MS has been the most often employed approach for analyzing significant mycotoxins in cereals out of all the typical procedures. Chromatographic approaches, particularly the LC/MS-MS technique, are indispensable for detecting various mycotoxins and food and food matrixes. Innovative techniques could prevent fungal proliferation in cereals, which can minimize mycotoxin contamination. Finally, minimizing the overall risk arising from mycotoxins is crucial to ensure public safety and safeguard the integrity of the food supply chain.

## CRediT authorship contribution statement

**Waqas Niaz:** Writing – review & editing, Writing – original draft, Validation, Software, Methodology, Formal analysis, Conceptualization. **Shahzad Z. Iqbal:** Writing – review & editing, Validation, Investigation, Formal analysis, Conceptualization. **Khurshid Ahmad:** Writing – review & editing, Visualization, Software, Investigation, Formal analysis. **Abdul Majid:** Writing – review & editing, Visualization, Validation, Software, Data curation. **Waqas Haider:** Writing – review & editing, Visualization, Software, Formal analysis. **Xianguo Li:** Writing – review & editing, Validation, Supervision, Project administration, Formal analysis, Conceptualization.

## Declaration of competing interest

The authors declare that they have no known competing financial interests or personal relationships that could have appeared to influence the work reported in this paper.

## Data Availability

Data will be made available on request.

## Appendix A Supplementary data

Supplementary material Image 1
